# Macrophage infectivity potentiator protein, a peptidyl prolyl *cis*-*trans* isomerase, essential for *Coxiella burnetii* growth and pathogenesis

**DOI:** 10.1371/journal.ppat.1011491

**Published:** 2023-07-03

**Authors:** Aleksandra W. Debowski, Nicole M. Bzdyl, David R. Thomas, Nichollas E. Scott, Christopher H. Jenkins, Jua Iwasaki, Emily A. Kibble, Chen Ai Khoo, Nicolas J. Scheuplein, Pamela M. Seibel, Theresa Lohr, Georgie Metters, Charles S. Bond, Isobel H. Norville, Keith A. Stubbs, Nicholas J. Harmer, Ulrike Holzgrabe, Hayley J. Newton, Mitali Sarkar-Tyson

**Affiliations:** 1 Marshall Centre for Infectious Disease Research and Training, School of Biomedical Sciences, The University of Western Australia, Nedlands, Western Australia, Australia; 2 School of Molecular Sciences, The University of Western Australia, Crawley, Western Australia, Australia; 3 Department of Microbiology and Immunology, Peter Doherty Institute for Infection and Immunity, The University of Melbourne, Melbourne, Australia; 4 Infection and Immunity Program, Department of Microbiology, Monash Biomedicine Discovery Institute, Monash University, Clayton, Australia; 5 Defence Science and Technology Laboratory, Porton Down, Salisbury, United Kingdom; 6 Wesfarmers Centre for Vaccines and Infectious Diseases, Telethon Kids Institute, University of Western Australia, Nedlands, Western Australia, Australia; 7 Centre for Child Health Research, University of Western Australia, Perth, Western Australia, Australia; 8 School of Veterinary and Life Sciences, Murdoch University, Perth, WA, Australia; 9 DMTC Limited, Level 1, Kew, Australia; 10 Institute of Pharmacy and Food Chemistry, University of Würzburg, Am Hubland, Würzburg, Germany; 11 Department of Biosciences, University of Exeter, Geoffrey Pope Building, Stocker Road, Exeter, United Kingdom; 12 Living Systems Institute, Stocker Road Exeter, United Kingdom; Universidade de São Paulo: Universidade de Sao Paulo, BRAZIL

## Abstract

*Coxiella burnetii* is a Gram-negative intracellular pathogen that causes the debilitating disease Q fever, which affects both animals and humans. The only available human vaccine, Q-Vax, is effective but has a high risk of severe adverse reactions, limiting its use as a countermeasure to contain outbreaks. Therefore, it is essential to identify new drug targets to treat this infection. Macrophage infectivity potentiator (Mip) proteins catalyse the folding of proline-containing proteins through their peptidyl prolyl *cis*-*trans* isomerase (PPIase) activity and have been shown to play an important role in the virulence of several pathogenic bacteria. To date the role of the Mip protein in *C*. *burnetii* pathogenesis has not been investigated. This study demonstrates that *Cb*Mip is likely to be an essential protein in *C*. *burnetii*. The pipecolic acid derived compounds, SF235 and AN296, which have shown utility in targeting other Mip proteins from pathogenic bacteria, demonstrate inhibitory activities against *Cb*Mip. These compounds were found to significantly inhibit intracellular replication of *C*. *burnetii* in both HeLa and THP-1 cells. Furthermore, SF235 and AN296 were also found to exhibit antibiotic properties against both the virulent (Phase I) and avirulent (Phase II) forms of *C*. *burnetii* Nine Mile Strain in axenic culture. Comparative proteomics, in the presence of AN296, revealed alterations in stress responses with H_2_O_2_ sensitivity assays validating that Mip inhibition increases the sensitivity of *C*. *burnetii* to oxidative stress. In addition, SF235 and AN296 were effective *in vivo* and significantly improved the survival of *Galleria mellonella* infected with *C*. *burnetii*. These results suggest that unlike in other bacteria, Mip in *C*. *burnetii* is required for replication and that the development of more potent inhibitors against *Cb*Mip is warranted and offer potential as novel therapeutics against this pathogen.

## Author summary

The bacterium *Coxiella burnetii* is a highly infectious organism that is found worldwide and can cause debilitating disease in both animals and humans. Treatment of chronic infections remains very difficult and therefore it is essential to identify new drug targets to treat this infection. The Macrophage infectivity potentiator (Mip) protein has been reported as an attractive anti-virulence target in several pathogenic bacteria but its role has not been investigated in *C*. *burnetii*. This study demonstrates that inhibitors of the Mip protein from *C*. *burnetii* reduce replication inside host cells, increase the sensitivity of the bacteria to oxidative stress, and exhibit antibiotic properties against both the virulent and avirulent forms of *C*. *burnetii*. Furthermore, using a moth larvae infection model, the inhibitors demonstrated protective activity. These results suggest that, unlike other studied bacterial pathogens, *C*. *burnetii* requires Mip for replication, and that the further development of potent inhibitors against Mip offer potential as novel therapeutics to treat this infection.

## Introduction

Q fever is a worldwide zoonotic disease caused by the bacterial pathogen *Coxiella burnetii*. The bacterium is highly infectious, with the infectious dose for humans estimated to be as low as 1–10 organisms [1]. Infection with *C*. *burnetii* can be asymptomatic and self-limiting. However, approximately 40% of infected individuals present with a severe flu-like illness lasting several weeks. In a minority of cases (~2%) acute infection can develop into persistent focal infections that can lead to serious health complications including endocarditis and vascular infections that will ultimately result in death if left untreated [2]. Treatment of acute infection is usually successful, however the two week recommended doxycycline treatment for acute disease [3,4] is sometimes poorly tolerated [2,5,6]. Complicating this treatment is that diagnosis and clearance of persistent infections is difficult [4,7,8]. Once persistent *C*. *burnetii* infection is diagnosed, the prescribed treatment is a combination of doxycycline and hydroxychloroquine lasting 18 or more months. Furthermore, approximately 20% of patients who recover from acute infection suffer for years from chronic fatigue, a condition known as Q fever fatigue syndrome that can severely impact their quality of life [9–12]. An effective human vaccine is available, (Q-VAX, Seqirus Australia), but it is only licenced in Australia and requires pre-vaccination screening as there are severe adverse reactions to the vaccine if the individual has immunity as a result of previous exposure to *C*. *burnetii* [13]. This severely limits the use of Q-VAX in the event of an outbreak. Consequently, there is a need to identify new approaches to treat this infection. This can be achieved through gaining a better understanding of both the bacterium’s biology and how it causes disease.

*C*. *burnetii* is a Gram-negative obligate intracellular pathogen with a biphasic lifecycle. The organism is shed into the environment by ruminant hosts, such as goats and sheep, in faeces, urine, milk and especially in birth products. The metabolically dormant and highly resilient small cell variant (SCV) of *C*. *burnetii* can persist from months to years in the environment [2,14,15]. Upon inhalation of contaminated aerosols, the SCVs are phagocytosed by resident alveolar macrophages and are trafficked through the endocytic pathway. Acidification of the phagocytic vesicle upon lysosomal fusion triggers transition of the SCV into the metabolically active large cell variant (LCV). This activates the Type IV Secretion System (T4SS) [16,17], which is functionally analogous to the Dot/Icm T4SS of *Legionella pneumophila* [18,19]. Over 130 T4SS effectors have been identified and they act to subvert multiple host pathways. Of particular importance are autophagy and vesicle trafficking, making the phagolysosome compartment, now termed a *Coxiella*-containing vacuole (CCV), permissive for *C*. *burnetii* replication [20–22]. The mature CCV is highly fusogenic, expanding to form one large CCV that can occupy almost the entire cell and support bacterial replication. As the CCV fills with bacteria, the LCVs transition back to the more stable SCVs which are then eventually released by a poorly defined egress [23]. During the replication process, *C*. *burnetii* also uses T4SS effector proteins to inhibit programmed cell death, one of several host defence mechanisms used to control pathogen replication [24–26]. In addition to the T4SS, *C*. *burnetii* has several other strategies to counteract host defence mechanisms. The bacteria are able to withstand the acidic conditions within the lysosome and resist the action of cationic peptides and lysosomal hydrolases [27]. These organisms are also able to resist macrophage killing mechanisms that involve the release of highly reactive oxidative species. *C*. *burnetii* suppress oxidative bursts [28], by expressing radical degrading enzymes and by expressing multiple DNA repair enzymes that serve to maintain genome integrity [29–31].

A major virulence associated protein that has been shown to be important in the pathogenesis of several Gram-negative pathogens is the Macrophage infectivity potentiator (Mip) protein [32,33]. Mip proteins belong to the peptidyl-prolyl *cis*-*trans* isomerase (PPIase) superfamily of proteins, also known as immunophilins, which catalyse the slow *cis*-*trans* isomerization of prolyl bonds and thereby increase the rate of protein folding. Gene deletion of *mip* in *Legionella* spp. significantly reduces the invasion and intracellular replication in human phagocytic cell lines and amoebae [34–39]. Significantly, Mip is essential for full virulence *in vivo*, with the *L*. *pneumophila mip* mutant reported to have reduced survival in a guinea pig infection model [40–42]. A similar report supporting the role of Mip in virulence is demonstrated in *Burkholderia pseudomallei*, where the loss of Mip results in decreased invasion and survival in J774A.1 macrophages and attenuation in the BALB/c mouse infection model [43].

In recent years immunophilin proteins have received considerable attention as druggable targets [44–47] and consequently there has been increased interest in targeting Mip as a therapeutic option against pathogenic bacteria [32,48–51]. Mip belongs to the FK506 binding proteins (FKBPs) subclass of the immunophilin superfamily, and like other FKBPs, Mip is inhibited by the macrolide antibiotics FK506 and rapamycin. However, the immunosuppressive activity of these compounds makes them unacceptable for use as therapeutic interventions [32]. Several recent publications have reported the synthesis and *in vitro* testing of Mip inhibitors which contain the important inhibitory component, pipecolic acid, found in FK506 and rapamycin, but lack their immunosuppressive properties. Compounds active against Mips from *Chlamydia trachomatis*, *Neisseria gonorrhoeae*, *Neisseria meningitidis*, and *B*. *pseudomallei* have been described, with some showing anti-virulence effects against these organisms [48,50,52–54]. More recently, a further refined subset of pipecolic acid based Mip inhibitors has been developed. These inhibitors have broad spectrum activity against Mip from multiple pathogens including *B*. *pseudomallei*, *N*. *meningitidis*, *Klebsiella pneumoniae* and the parasite *Leishmania major* [55].

The Mip homologue in *C*. *burnetii* (*Cb*Mip) was first described and characterized by Mo *et al*. [56]. The *Cb*Mip catalytic domain has a high degree of sequence similarity to characterized Mip proteins of other intracellular Gram-negative pathogens including *L*. *pneumophila*, *B*. *pseudomallei* and *C*. *trachomatis* (S1 Fig) [35,43,56,57]. Consequently, *Cb*Mip has been considered a virulence factor of *C*. *burnetii* [37,58,59]. However, the role of *Cb*Mip in *C*. *burnetii* pathogenesis has not been directly investigated. In this study, using both the virulent and avirulent forms of *C*. *burnetii* Nine Mile RSA439 (*C*. *burnetii* NMI and *C*. *burnetii* NMII, respectively), the role of the *Cb*Mip in *C*. *burnetii* pathogenesis, including intracellular replication and growth in axenic media, was investigated. The potential for using inhibitors targeting *Cb*Mip to modulate *C*. *burnetii* virulence in *Galleria mellonella* larvae was also explored. The results suggest that unlike in other bacteria, Mip in *C*. *burnetii* is required for replication and that inhibitors against *Cb*Mip offer potential as novel therapeutics against the bacterium.

## Results

### Recombinant *Cb*Mip PPIase activity is inhibited by SF235 and ANCH37

**Fig 1:**
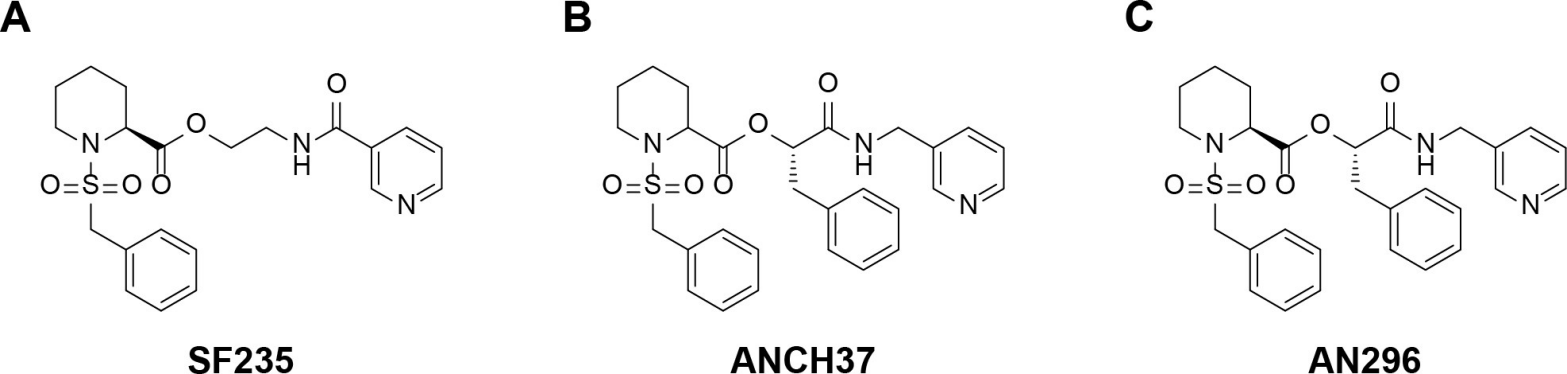
Structures of pipecolic acid-based Mip inhibitors, SF235, ANCH37 and AN296. The synthesis of these inhibitors has been described elsewhere [50,61]).

To first investigate if *Cb*Mip has a role in virulence of *C*. *burnetii*, validated Mip inhibitors SF235, ANCH37 and AN296 (Fig 1) were utilised, following approaches used to study the role of Mip in another obligate intracellular pathogen, *C*. *trachomatis* [54,60]. Although SF235 and ANCH37 are pipecolic acid derivatives that have been optimised against Mip from *B*. *pseudomallei* they also display activity against Mips from other bacterial species [50,55,61]. ANCH37 (Fig 1B), like SF235, contains the pipecolic sulphonamide and an amide-based linker to a pyridyl unit, but also contains an additional benzyl moiety which has been shown to increase potency. Initially, the activity of SF235 and ANCH37 against recombinantly expressed *Cb*Mip was assessed using a high throughput peptidyl-prolyl *cis*-*trans* isomerase assay [62]. Both compounds were found to be inhibitors of *Cb*Mip with *K*_*i*_ values of 18±4 μM for SF235 and 4.6±0.9 μM for ANCH37 (S2 Fig). ANCH37 is a racemic mixture consisting of two stereoisomers. The most active compound within the mixture, AN296 (Fig 1C), was prepared [61] and used in the following studies.

### *Cb*Mip inhibitors SF235 and AN296 reduce intracellular replication of *C*. *burnetii*

**Fig 2:**
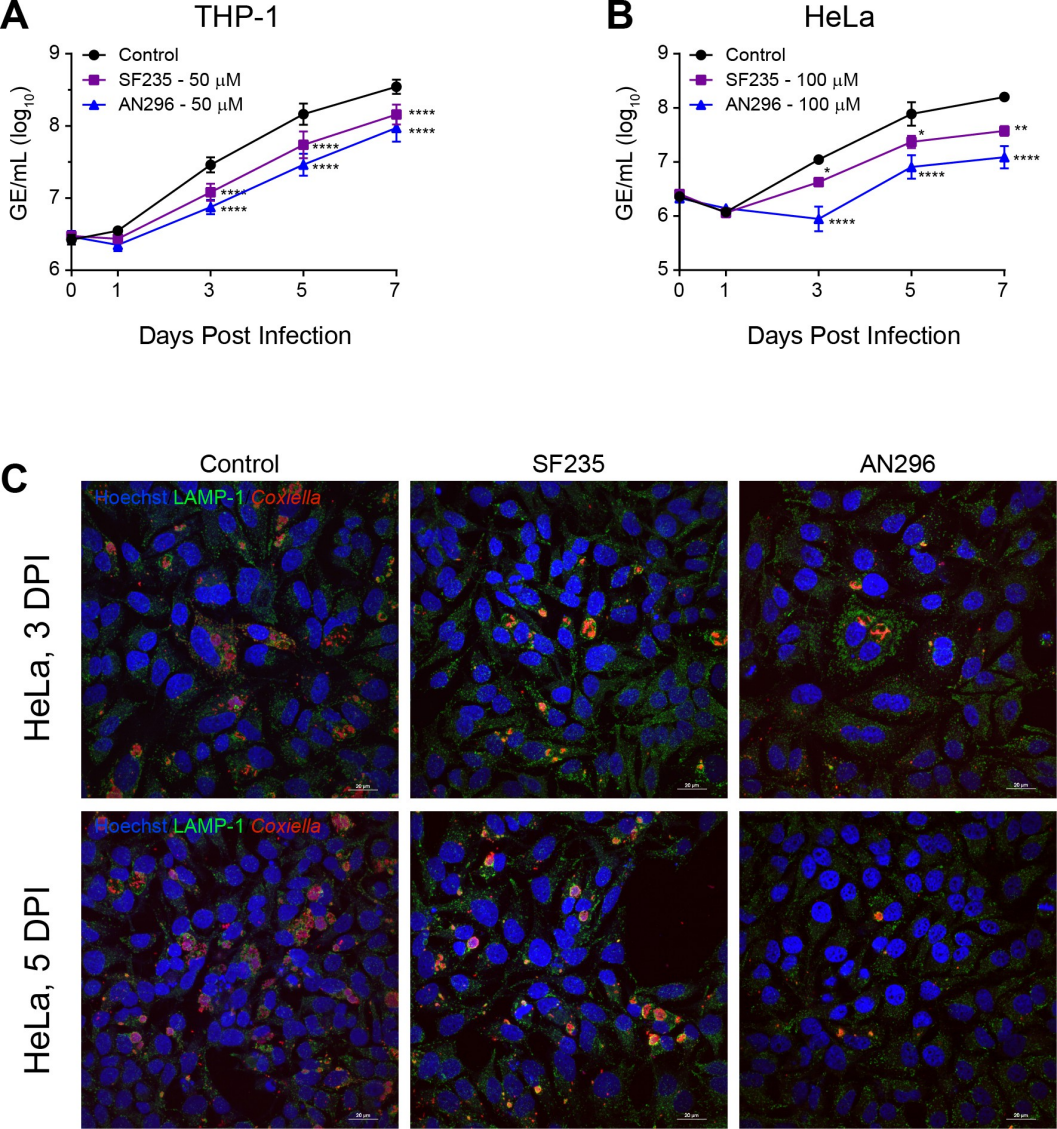
Inhibitors of *Cb*Mip affect intracellular replication of *C*. *burnetii*. (A and B) Intracellular replication of *C*. *burnetii* NMII in the presence of *Cb*Mip inhibitor SF235 (purple square), AN296 (blue triangle) or vehicle control (black circle). (A) THP-1 cells with 50 μM of inhibitor (*n* ═ 6) and (B) HeLa cells with 100 μM of inhibitor (*n* ═ 3). Error bars represent standard error of the mean. *, *p* < 0.05: **, *p* < 0.01; ****, *p* < 0.0001. *p* values were determined using two-way ANOVA, followed by Dunnett’s multiple comparison post-test. (C) Representative confocal immunofluorescence (IF) images at 3 and 5 days post-infection (DPI) for HeLa cells. Cells were stained with anti-LAMP1 (green), anti-*Coxiella* (red), and Hoechst 33258 (blue). Scale bar ═ 20 μm.

Having confirmed that SF235 and AN296 are potent inhibitors of *Cb*Mip, their effect on the intracellular replication of *C*. *burnetii* was investigated. *C*. *burnetii* NMII was first pretreated with SF235 or AN296 for 1 h, to facilitate complete inhibition of *Cb*Mip including any enzyme that may be localized in the cytoplasm [63], and used to infect differentiated THP-1 macrophage cells or HeLa epithelial cervical cancer cells. *C*. *burnetii* replication was then monitored by measuring genome equivalents (GE) over 7 days. In the presence of 50 μM AN296, intracellular replication of *C*. *burnetii* NMII within THP-1 cells was significantly inhibited (Fig 2A), resulting in a 74%, 80% and 73% (*p* < 0.0001) reduction in *C*. *burnetii* NMII replication compared to the control, 3, 5, and 7 days post infection, respectively. Although slightly less effective, similar results were observed for SF235, where inhibitor treatment (50 μM) resulted in a 58%, 62% and 59% (*p* < 0.0001) reduction in *C*. *burnetii* NMII replication 3, 5, and 7 days post infection, respectively. *C*. *burnetii* NMII replication was also significantly reduced in HeLa cells when cultures were treated with higher concentrations of Mip inhibitor. In the presence of 100 μM AN296, intracellular replication of *C*. *burnetii* NMII was significantly inhibited in HeLa cells, resulting in a 92%, 90% and 92% (*p* < 0.0001) reduction in *C*. *burnetii* NMII replication 3, 5, and 7 days post infection, respectively (Fig 2B). Furthermore, compared to the untreated control, treatment with 100 μM AN296 appeared to delay the replication kinetics of *C*. *burnetii* NMII within HeLa cells by two days. The effect on *C*. *burnetii* NMII replication in HeLa cells treated with 100 μM of SF235 was less pronounced but still significant and resulted in a 62%, 70% (*p* < 0.05) and 76% (*p* < 0.01) reduction in *C*. *burnetii* NMII replication at 3, 5, and 7 days post infection compared to control. HeLa cells were also analysed by immunofluorescence staining at day 3 and 5 post infection and interestingly showed that CCVs were smaller in the presence of SF235 or AN296 compared to the control (Fig 2C). The infection experiments were repeated in THP-1 cells without pre-exposing *C*. *burnetii* to the *Cb*Mip inhibitors. Addition of 50 μM AN296 after the initial 4 h infection period also significantly inhibited *C*. *burnetii* NMII replication within THP-1 cells (S3 Fig), resulting in a 71%, 69% and 63% (*p* < 0.0001) reduction in *C*. *burnetii* NMII replication at 3, 5, and 7 days post infection, respectively. The antibiotic chloramphenicol (31 μM), a potent inhibitor of bacterial protein synthesis and *C*. *burnetii* replication [64], was used as a positive control. As expected, addition of chloramphenicol completely inhibited *C*. *burnetii* NMII replication within THP-1 cells (S3 Fig). Both SF235 and AN296 were also considered non-toxic to the cells over the course of the infection assay at the concentrations used in each cell-based assay (S4 Fig).

### The potential essential nature of Mip in *C*. *burnetii*

**Fig 3:**
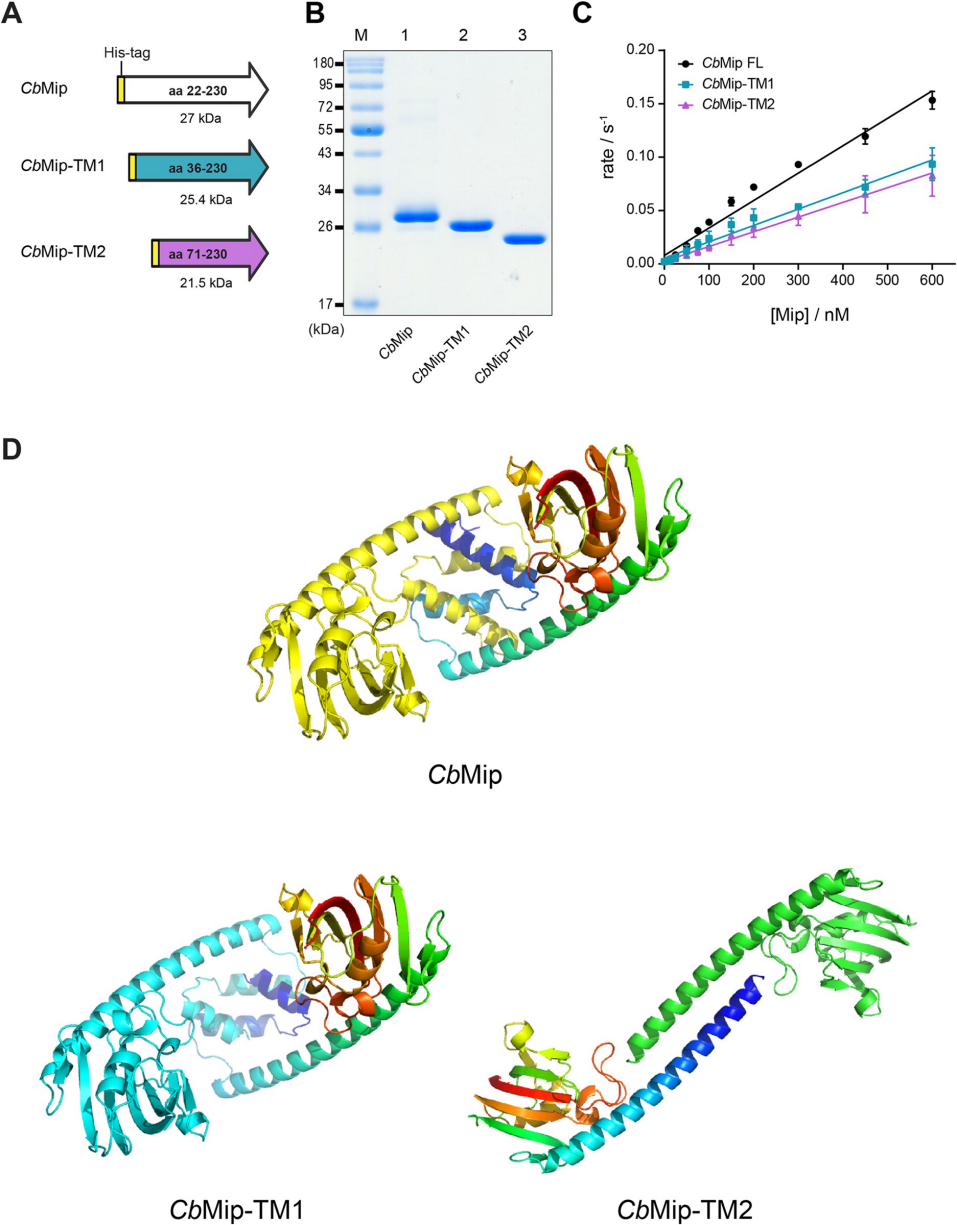
Activity of truncated *Cb*Mip proteins. (A) Schematic diagram of recombinantly expressed truncated *Cb*Mip variants. *Cb*Mip-TM1 and *Cb*Mip-TM2 are truncated *Cb*Mip variants that are predicted to be expressed by the *C*. *burnetii cbmip* transposon mutants. All truncated *Cb*Mip variants contain an N-terminal His-tag like the full length *Cb*Mip to facilitate protein purification. (B) Representative Coomassie stained SDS-PAGE gel of purified recombinant truncated *Cb*Mip proteins. *Cb*Mip proteins were purified by nickel affinity chromatography followed by size exclusion chromatography (SEC) purification. Lane M, Prestained Protein Standard (New England Biolabs) was included for approximation of molecular weight. Lane 1, the expected molecular weight of full length *Cb*Mip on a 12% SDS-PAGE gel is 27 kDa; Lane 2, *Cb*Mip-TM1 is 25.4 kDa; Lane 3, *Cb*Mip-TM2 is 21.5 kDa. (C) *k*_*cat*_*/K*_*M*_ of full length and truncated variants of *Cb*Mip. The rate of each Mip protein was tested at a range of concentrations and each concentration was tested three times. The *k*_*cat*_*/K*_*M*_ values were determined to be 2.57 ± 0.08 x 10^5^ s^-1^ M^-1^, 1.53 ± 0.06 x 10^5^ s^-1^ M^-1^, and 1.38 ± 0.07 x 10^5^ s^-1^ M^-1^ for CbMip, and the truncates *Cb*Mip-TM1 and *Cb*Mip-TM2, respectively. Error bars show standard errors. (D) The structure of *Cb*Mip and the truncated variants *Cb*Mip-TM1 and *Cb*Mip-TM2 were predicted using AlphaFold2, using the multimer prediction option to model dimers. Structures are presented in cartoon format with one protomer in a single colour (yellow for *Cb*Mip, cyan for *Cb*Mip-TM1, green for *Cb*Mip-TM2) and the other as a rainbow from the N-terminus (blue) to C-terminus (red). Five replicates of the *Cb*Mip and *Cb*Mip-TM1 models gave very similar structures and dimer architectures. In contrast, five models of *Cb*Mip-TM2 gave very similar structures for the FKBP domain, but different architectures for a dimer, indicating that this truncated protein is likely to be monomeric. Images generated using PyMOL v. 2.5.3 (Schrödinger).

The inhibitory effect of AN296 and SF235 on *C*. *burnetii* intracellular replication suggested that Mip may be essential in *C*. *burnetii*. To address this directly, attempts were made to delete the *mip* gene (*cbu*0630, *cbmip*) from *C*. *burnetii* NMII. However, despite obtaining integration of the pJC-CAT suicide plasmid, all attempts to replace *cbmip* with a kanamycin resistance cassette were unsuccessful. Furthermore, supporting this hypothesis that *cbmip* is an essential gene, a transposon insertion library of *C*. *burnetii* NMII previously generated by Metters *et al*. [65] was interrogated for the presence of *cbmip* insertion mutants. Analysis of the sequencing data revealed two *cbmip* transposon mutants (S5 Fig), the same number of mutants that would have been expected by chance given the library depth (personal comms; Georgie Metters). Interestingly, both transposon insertion sites were located within the first 200-bp of *cbmip* and, in both instances, the first ATG sequence following the insertion site was in-frame with the original gene sequence leaving an intact catalytic domain (S5C Fig). The predicted *Cb*Mip peptide sequences from these *cbmip* transposon mutant variants were recombinantly expressed (Fig 3A and 3B) and shown to have retained 50–60% of their PPIase activity (Fig 3C). The *k*_*cat*_*/K*_*M*_ values were determined to be, 1.53 ± 0.06 x 10^5^ s^-1^ M^-1^, and 1.38 ± 0.07 x 10^5^ s^-1^ M^-1^ for the truncated proteins, *Cb*Mip-TM1 and *Cb*Mip-TM2, respectively as compared to 2.57 ± 0.08 x 10^5^ s^-1^ M^-1^ for *Cb*Mip. Modelling of *Cb*Mip-TM1 and *Cb*Mip-TM2 using AlphaFold [66] (Fig 3D) demonstrated that the PPIase domain is likely intact in both truncated proteins, and that *Cb*Mip-TM1 may retain dimer formation. However, *Cb*Mip-TM2 lacks most of the dimerization domain, and several alternative dimer architectures were modelled. These data suggest that for both *cbmip* transposon mutants identified in the Metters *et al*. library, it is very likely that a truncated, but functional, *Cb*Mip protein is expressed by these mutants and therefore they do not have a true *mip* gene disruption, suggesting that *Cb*Mip may be essential for *C*. *burnetii* growth.

### *Cb*Mip inhibitors reduce *C*. *burnetii* NMII growth in axenic culture in a dose dependant manner

**Fig 4:**
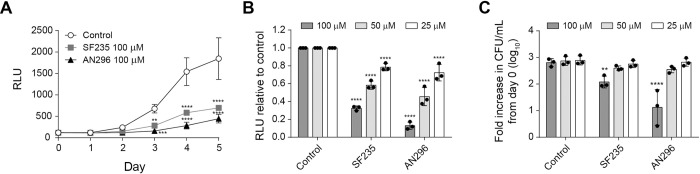
Targeted inhibition of *Cb*Mip reduces *C*. *burnetii* replication in axenic media in a dose-dependent manner. (A) Bioluminescent was measured as an indicator of *C*. *burnetii*-lux replication. *C*. *burnetii*-lux was inoculated at a concentration of 1 × 10^6^ GE/mL into ACCM-2 media with 100 μM of *Cb*Mip inhibitors SF235 (grey square), AN296 (closed triangle) or vehicle control (open circle), and growth was monitored over 5 days. Data is presented as RLU (relative light units) with error bars representing the standard deviation (SD) from three independent experiments. (B) *C*. *burnetii*-lux replication over 5 days in the presence of 25 μM, 50 μM or 100 μM of SF235 or AN296. Data is presented as RLU relative to the vehicle control with error bars representing SD from at least three independent experiments. (C) Colony forming units per mL was determined for *C*. *burnetii* NMII after 4 days of growth in the presence of 25 μM, 50 μM or 100 μM of SF235 or AN296. Data is presented as the fold increase in CFU/mL relative to day 0 with error bars representing the SD from at least three independent experiments. *, *p* < 0.05; **, *p* < 0.01; ***, *p* < 0.001; ****, *p* < 0.0001. *p* values were determined using two-way ANOVA followed by Dunnett’s multiple comparison post-test.

To investigate whether Mip inhibition directly affected *C*. *burnetii* growth, a luciferase-expressing derivative of *C*. *burnetii* NMII, *C*. *burnetii*-lux, was evaluated in axenic media in the presence of different concentrations of SF235 and AN296. In the first instance, due to the pH of the axenic media (4.75) and that the compounds both contain ester linkages, the stability of the compounds was investigated by HPLC chromatography. Using AN296 as an exemplar, it was found to be stable under the acidic conditions of the assay over the entire 7-day period studied (S6 Fig). Proceeding with the axenic experiments, luciferase activity of *C*. *burnetii*-lux was inhibited in a dose-dependent manner compared to the control (Figs 4A and S7). Following normalization to DMSO, the presence of SF235 reduced *C*. *burnetii*-lux bioluminescence by 68% and 42% (*p* < 0.0001) at 100 μM and 50 μM, respectively. Treatment with AN296 reduced bioluminescence by 87% and 55% (*p* < 0.0001) at 100 μM and 50 μM, respectively (Fig 4B). To confirm that the compounds were affecting *C*. *burnetii* growth and not simply preventing proper folding and function of the luciferase, the experiment was repeated using wild-type *C*. *burnetii* NMII and colony forming units (CFU) were enumerated after 4 days of culture. The fold-increase in *C*. *burnetii* NMII CFU/mL (log_10_) compared to day 0, in the presence of 100 μM or 50 μM of *Cb*Mip inhibitor was 19% (*p* < 0.01) and 54% of the control, respectively, for SF235, and 2.1% (*p* < 0.0001) and 48% of the control, respectively, for AN296 (Fig 4C). This data demonstrates that SF235 and AN296 directly limit the ability of *C*. *burnetii* to replicate in axenic media.

**Fig 5:**
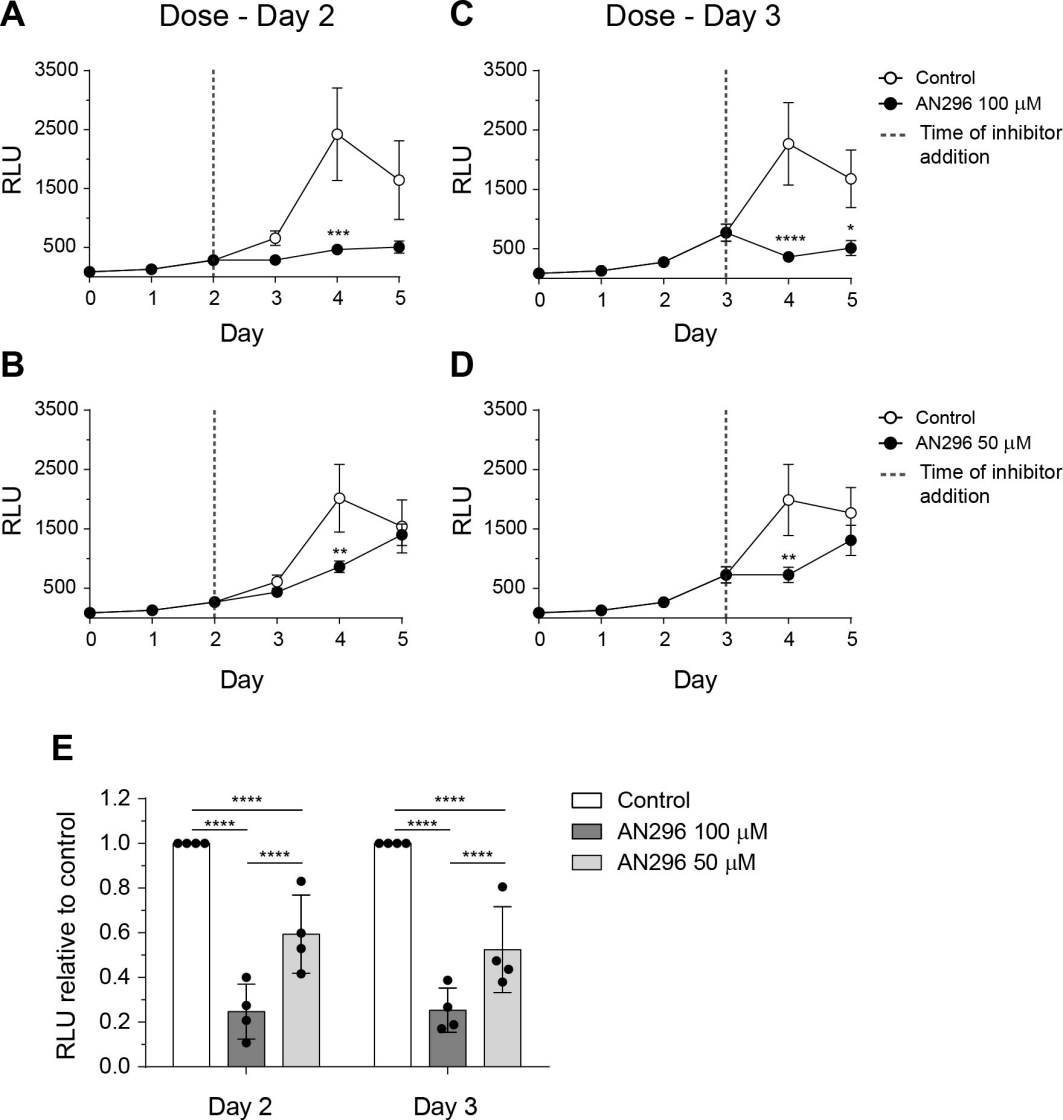
Delayed dosing with AN296 impairs *C*. *burnetii* replication in axenic media. Bioluminescence was measured as an indicator of *C*. *burnetii*-lux replication. The strain was inoculated at a concentration of 1 × 10^6^ GE/mL into ACCM-2 media and growth was monitored over 5 days. Cultures were dosed with (A+B) 100 μM or (C+D) 50 μM of AN296 (closed circle) or vehicle control (open circle) on (A+C) day 2 or (B+D) day 3 of the growth curve. Data is presented as RLU (relative light units) with error bars represent standard error of the mean from four independent experiments. *, *p* < 0.05; **, *p* < 0.01; ***, *p* < 0.001; ****, *p* < 0.0001. *p* values were determined using two-way ANOVA, followed by Sidak’s multiple comparison post-test. (E) *C*. *burnetii*-lux replication after dosing with 50 μM or 100 μM of AN296 on day 2 or day 3 of the 5 day growth curve. Data is presented as RLU relative to the control with error bars representing SD from at least four independent experiments. ****, *p* < 0.0001. *p* values were determined using two-way ANOVA, followed by Tukey’s multiple comparison post-test.

The potential of Mip inhibitors to act on exponentially growing *C*. *burnetii* cultures was also investigated using the most effective compound AN296 (Fig 5). Addition of AN296 to the culture on either day 2 (start of logarithmic growth) (Fig 5A and 5B) or day 3 (mid-logarithmic growth) (Fig 5C and 5D) [67] resulted in a significant decrease in bioluminescence. Following normalization to the control, addition of AN296 on day 2 reduced *C*. *burnetii*-lux bioluminescence by 75% and 41% (*p* < 0.0001) at 100 μM and 50 μM (Fig 5E), respectively. Similarly, addition of AN296 on day 3 reduced bioluminescence by 75% and 48% (*p* < 0.0001) at 100 μM and 50 μM (Fig 5E), respectively. These data demonstrate that AN296 is effective at suppressing the growth of both starter cultures that are predominantly composed of *C*. *burnetii* SCVs and actively growing cultures which contain *C*. *burnetii* cells that are predominantly LCVs [68]. Together these data suggest that the presence of *Cb*Mip inhibitors perturb the ability of *C*. *burnetii* to replicate both intracellularly and in axenic media.

### Inhibition of *Cb*Mip induces changes on the *C*. *burnetii* proteome

To elucidate the phenotypic effects induced by the presence of *Cb*Mip inhibitors and the inability to generate a *cbmip* mutant in *C*. *burnetii*, a proteomics approach was used to gain insight into the cellular processes and specific proteins impacted by the inhibition of *Cb*Mip. Mid-log phase (day 3) grown *C*. *burnetii* NMII were exposed to 100 μM of AN296 for 24 h and compared to control treatments. This revealed only modest changes across the proteome with only 18 proteins significantly altered in response to AN296 treatment (Table 1). Analysis of the KEGG Orthology (KO) categorization associated with these proteins revealed that approximately half of the proteins that underwent significant changes were either unclassified or hypothetical proteins (11/18). Of note however is that two of the proteins that have increased relative abundance, CBU1576 and CBU1686, have been previously identified as putative T4SS effector proteins [69–71]. Interestingly, four other proteins with an increase in relative abundance (RecG, RecQ, PriA and UvrD) were classified as being involved in DNA replication, recombination, and repair. The increased relative abundance of several DNA repair enzymes suggests that *C*. *burnetii* NMII may be more sensitive to oxidative stress when grown in the presence of AN296.

**Table 1:** List of proteins that underwent statistically significant alterations within the proteome of *C*. *burnetii* NMII grown in axenic cultures after 24 h of exposure to AN296 (100 μM). White cells indicate relative increase, grey cells indicate relative decrease compared to the culture treated with vehicle control (DMSO).

KEGG Orthology (KO)^1^	CBU number	Gene name	Gene product	Fold change (Log_2_)	-Log_10_ Student’s *t*-test *p*-value
***Metabolism***					
Amino acid	CBU1839		Aminobutyraldehyde dehydrogenase	1.04042	3.22445
LPS biosynthesis	CBU0142	*lpxC*	UDP-3-O-acyl-N-acetylglucosamine deacetylase	1.24371	1.61264
***Genetic information processing***					
DNA repair and recombination	CBU0305	*recG*	ATP-dependent DNA helicase RecG	1.38111	4.54836
	CBU0472	*recQ*	ATP-dependent DNA helicase	1.29853	2.68489
	CBU1815	*priA*	Primosomal protein N’ (ATP-dependent helicase PriA)	1.31784	4.40603
	CBU2054	*uvrD*	DNA helicase II	1.69821	2.45767
***Signalling and cellular processes***					
Other	CBU1490	*higA*	Virulence-associated protein I	1.77358	1.92185
***Unclassified and Hypothetical proteins***					
	CBU0022		Hypothetical cytosolic protein	1.10539	2.00792
	CBU0941		Probable Fe(2+)-trafficking protein	1.04587	1.55144
	CBU0637		Coenzyme PQQ synthesis protein C	1.60759	3.47957
	CBU1576		Putative T4SS effector protein [71]	1.11642	2.11499
	CBU1686		Putative T4SS effector protein [69,70]	1.17901	2.98394
	CBU1753		Hypothetical cytosolic protein	1.23788	1.46301
	CBU2057		Hypothetical cytosolic protein	1.1392	3.73108
	CBU0089a		Uncharacterized protein	-2.31933	3.5896
	CBU0110		Hypothetical exported protein	-1.38947	2.67694
	CBU1095		Hypothetical exported protein	-1.05242	1.663
	CBU1308		Phosphohydrolase family protein	-1.25087	2.0132

^1^ Proteins that were determined to be differentially present by proteomics were manually curated using the Kyoto Encyclopaedia of Genes and Genomes (KEGG) database against the *Coxiella burnetii* RSA 493 genome (entry number T00124) and assigned a KEGG Orthology (KO). *C*. *burnetii* proteins were further clustered according to their predicted functions as reported by the KEGG database.

### *Cb*Mip inhibition increases *C*. *burnetii* sensitivity to oxidative stress

**Fig 6:**
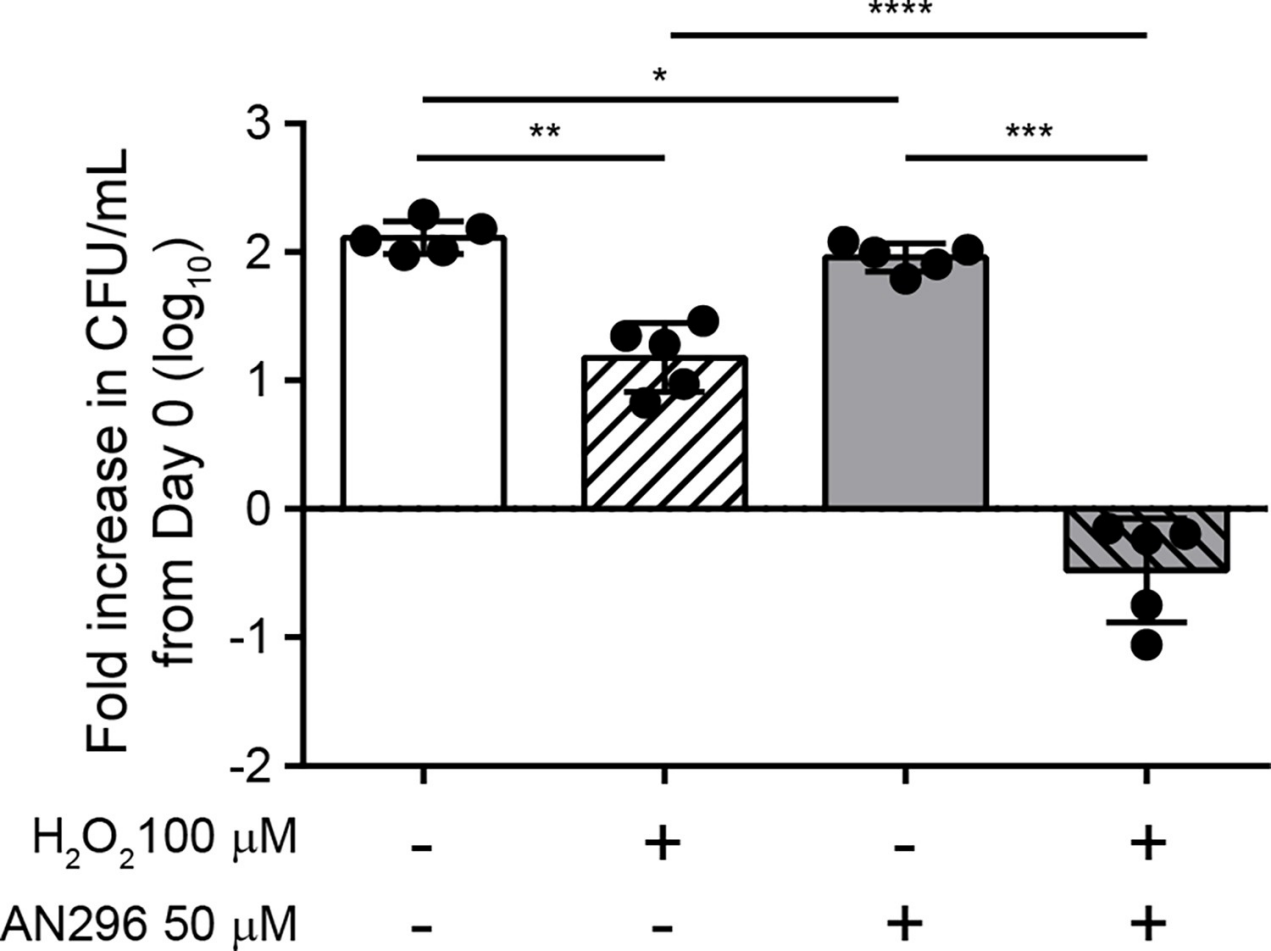
Inhibition of *Cb*Mip increases *C*. *burnetii* sensitivity to oxidative stress. *C*. *burnetii* NMII was grown in ACCM-2 media inoculated with either 50 μM AN296 or vehicle control, in the absence or presence of 100 μM H_2_O_2_. After 4 days of incubation, cultures were plated out to determine viable colony forming units/mL. Data is presented as the log_10_ fold increase in CFU/mL from day 0 with error bars representing SD from five independent experiments. *, *p* < 0.05; **, *p* < 0.01; ***, *p* < 0.001; ****, *p* < 0.0001. *p* values were determined using one-way ANOVA, followed by Tukey’s multiple comparison post-test.

To validate whether Mip inhibition in *C*. *burnetii* results in an increased susceptibility to oxidative stress, hydrogen peroxide sensitivity assays were performed. *C*. *burnetii* NMII was cultured in axenic media containing no or 100 μM H_2_O_2_ and treated with AN296 (50 μM) or DMSO as a control (Fig 6). Enumeration of bacteria after four days of incubation showed that the fold increase in *C*. *burnetii* NMII CFU/mL (log_10_) compared to day 0, was significantly lower for cultures treated with either 100 μM of H_2_O_2_ (*p* < 0.01) or 50 μM AN296 (*p* < 0.05), which was not unexpected. However, when *C*. *burnetii* NMII was exposed to the combined treatment of 100 μM H_2_O_2_ and 50 μM AN296 the bacteria failed to replicate in the culture conditions, and lost viability, with an average fold decrease in CFU/mL of 0.48±0.40 (*p* < 0.0001) (Fig 6). This result demonstrated that the presence of *Cb*Mip inhibitor dramatically increases the susceptibility of *C*. *burnetii* to oxidative stress.

### Inhibition of *Cb*Mip stops replication of virulent *C*. *burnetii* in axenic culture

**Fig 7:**
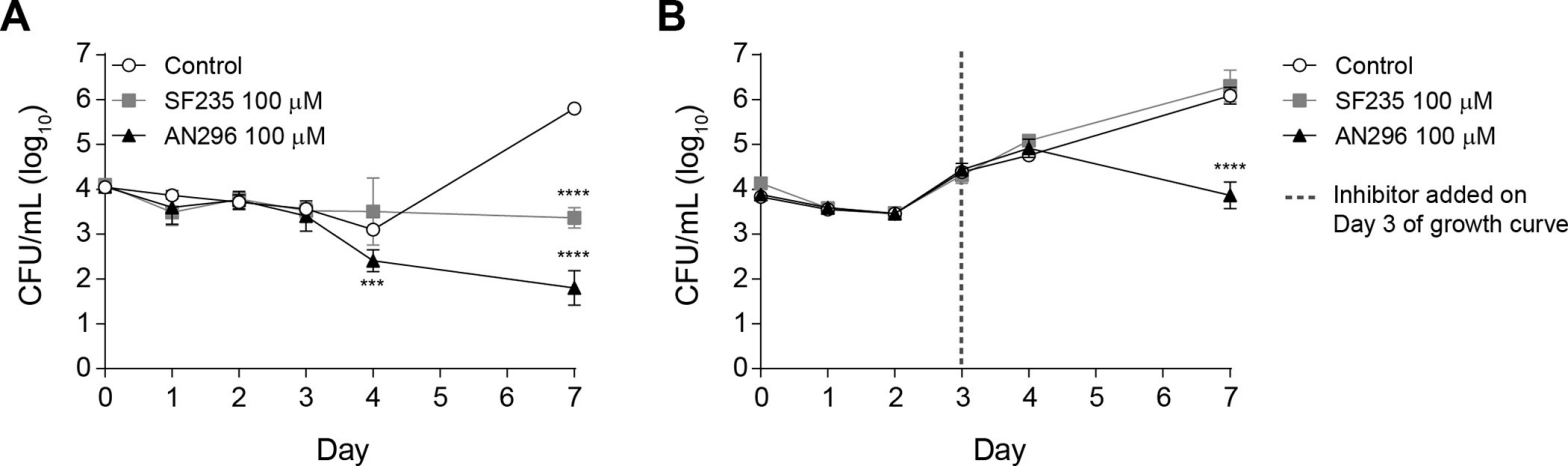
AN296 is highly potent against virulent *C*. *burnetii* NMI in axenic media. Growth curve of *C*. *burnetii* NMI in the presence of *Cb*Mip inhibitors. (A) *C*. *burnetii* NMI was inoculated at a concentration of 1 × 10^4^ CFU/mL into 5 mL of ACCM-2 media supplemented with 0.50 mM tryptophan and containing 100 μM of *Cb*Mip inhibitors, SF235 (grey square), AN296 (closed triangle), or vehicle control (open circle), and growth was monitored over 7 days by enumerating the number of colony forming units per mL in the culture on days 0, 1, 2, 3, 4 and 7. Data is presented as log_10_ CFU/mL with error bars representing the SD from at least three independent experiments. ***, *p* < 0.001; ****, *p* < 0.0001. *p* values were determined using two-way ANOVA followed by Dunnett’s multiple comparison post-test. (B) *C*. *burnetii* NMI was inoculated at a concentration of 1 × 10^4^ GE/mL into 5 mL of ACCM-2 media supplemented with 0.50 mM tryptophan. After 3 days, cultures were inoculated with 100 μM of *Cb*Mip inhibitors, SF235 (grey square), AN296 (closed triangle), or vehicle control (open circle). Growth was monitored over 7 days by enumerating the number of colony forming units per mL in the culture on days 0, 1, 2, 3, 4 and 7. Data is presented as log_10_ CFU/mL with error bars representing the SD from at least three independent experiments; ****, *p* < 0.0001. *p* values were determined using two-way ANOVA followed by Dunnett’s multiple comparison post-test.

For *Cb*Mip inhibitors to be therapeutically useful, they must be active against the virulent, phase I form of *C*. *burnetii*. Therefore, the activity of compounds AN296 and SF235 against the phase I parental strain *C*. *burnetii* NMI was investigated. The growth of *C*. *burnetii* NMI in axenic media while exposed to 100 μM of SF235 or AN296 versus a vehicle control was monitored over 7 days by enumerating CFU/mL for the first 4 days and then on day 7 (Fig 7A). *C*. *burnetii* NMI replication was significantly inhibited when grown in the presence of AN296 compared to the control culture at day 4 (*p* < 0.001) and 7 (*p* < 0.0001) (Fig 7A). Although slightly less effective, treatment with SF235 also significantly inhibited *C*. *burnetii* NMI replication compared to the control on day 7 (*p* < 0.0001). The inhibitors were also tested against *C*. *burnetii* NMI during later stages of growth (Fig 7B). Addition of 100 μM of SF235 to the culture on day 3 had no impact on the growth of *C*. *burnetii* NMI compared to the control. However, the addition of 100 μM of AN296 on day 3 significantly inhibited *C*. *burnetii* NMI growth compared to the control on day 7 (*p* < 0.0001*)*. Together these data indicate that SF235 and AN296 also directly impact on the ability of virulent *C*. *burnetii* NMI to replicate in axenic media. Given the observed antibacterial effect of AN296 on the growth of *C*. *burnetii* NMI, the minimal inhibitory concentration (MIC) and minimal bactericidal concentration (MBC) values were determined for SF235 and AN296 against both the NMI and NMII strains. After 6 days of incubation in ACCM-2 media, the MIC for AN296 was determined to be 100 μM and SF235 exhibited an MIC of 200 μM for both strains (Table 2). The MBCs for both these compounds was determined to be greater than the highest concentration tested (>400 μM). However, significantly fewer bacteria were recovered from cultures treated with 400 μM AN296 compared to cultures treated with SF235, and in both instances the recoverable CFU/mL was lower than those of the starting inoculums.

**Table 2:** Antibiotic efficacy of *Cb*Mip inhibitors for *C*. *burnetii* NMI and *C*. *burnetii* NMII^a^.

Compound	*C*. *burnetii* NMI		*C*. *burnetii* NMII	
	MIC^b^	MBC^c^	MIC^b^	MBC^c^
SF235	100–200 μM	> 200 μM	100–200 μM	> 400 μM
AN296	100 μM	> 200 μM	100 μM	> 400 μM

^a^MIC was defined as the lowest concentration of antibiotic required to inhibit bacterial growth in 6 day cultures; MBC was defined as the lowest concentration to give no visible growth when plated onto ACCM-2 agar and incubated for 7 days.

^b^Determined by OD in broth culture.

^c^Determined by CFU in broth culture.

### AN296 reduces intracellular replication of *C*. *burnetii* NMII during active infection

**Fig 8:**
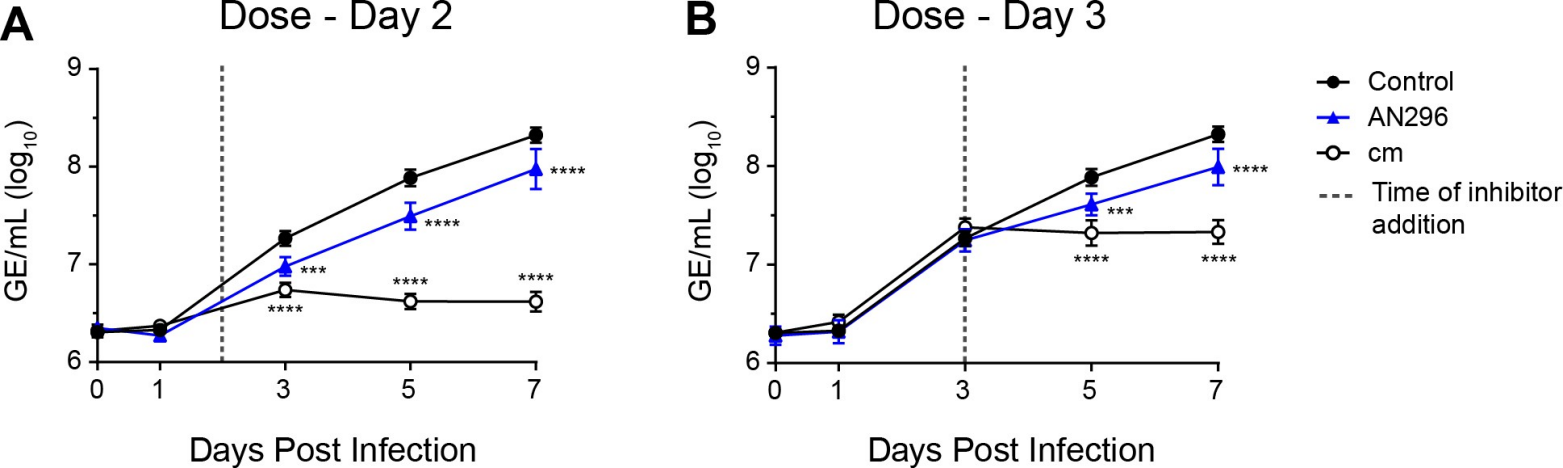
Delayed dosing with *Cb*Mip inhibitors also affects intracellular replication of *C*. *burnetii*. Intracellular replication of *C*. *burnetii* NMII in THP-1 cells in the presence of 50 μM AN296 (blue triangle), 31 μM chloramphenicol (Cm) (open circle) or control (closed circle), introduced at (A) day 2 or (B) day 3 of infection. Error bars represent standard error of the mean (*n* ═ 5). ***, *p* < 0.001; ****, *p* < 0.0001. *p* values were determined using two-way ANOVA, followed by Dunnett’s multiple comparison post-test.

Considering the potent effect these *Cb*Mip inhibitors had on *C*. *burnetii* growth, especially AN296, the therapeutic capacity of this compound was examined further by investigating the effect on *C*. *burnetii* NMII replication in THP-1 cells by administering the compound during active infection. Differentiated THP-1 cells were infected with *C*. *burnetii* NMII, and AN296 (50 μM) was added to the cell culture medium on day 2 or day 3 of infection. The antibiotic chloramphenicol (31 μM) was used as a positive control. Addition of AN296 even during later stages of *C*. *burnetii* NMII infection significantly reduced intracellular replication (Fig 8). Addition of AN296 on day 2 of infection resulted in 60% and 55% (*p* < 0.0001) reduction in *C*. *burnetii* NMII replication compared to the control at 5 and 7 days post infection, respectively (Fig 8A). Addition of AN296 on day 3 of infection resulted in a 47% (*p* < 0.001) and 53% (*p* < 0.0001) reduction in *C*. *burnetii* NMII replication at 5 and 7 days post infection, respectively (Fig 8B). As expected, addition of chloramphenicol, at day 2 or day 3 of infection stopped further *C*. *burnetii* NMII replication (Fig 8). This data shows that inhibitors of *Cb*Mip can be used to reduce the replication of *C*. *burnetii* NMII during active cell infection.

### AN296 and SF235 improve survival of *Galleria mellonella* larvae in *C*. *burnetii* infection model

**Fig 9:**
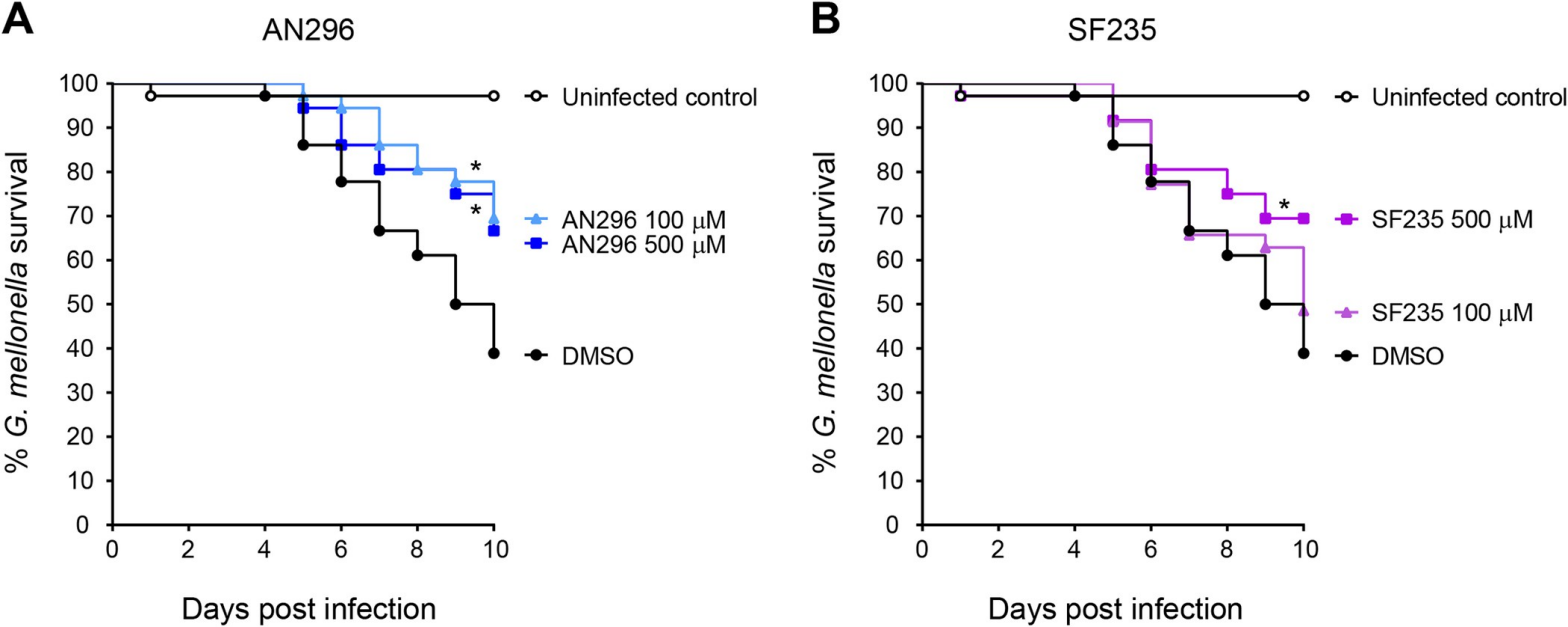
Administration of *Cb*Mip inhibitors improves survival of *G*. *mellonella* larvae in *C*. *burnetii* infection model. *C*. *burnetii* NMII were incubated in PBS containing 1% DMSO and 0, 100 μM or 500 μM of (A) AN296 or (B) SF235 for 1 h at 37°C prior to challenge of *G*. *mellonella* larva (*n* ═ 12 per group) with the mixture. Larvae were challenged with a single dose of 10^6^ GE of *C*. *burnetii* NMII with or without *Cb*Mip inhibitors and survival was assessed every 24 h for 10 days. Uninfected controls received PBS containing 1% DMSO. Kaplan-Meier survival curves were determined from three independent experiments. Data was analysed using the log-rank (Mantel-Cox) test with Bonferroni correction for multiple comparisons with a significance level of 0.05.

To investigate the efficacy of the Mip compounds *in vivo*, the *Galleria mellonella* larva infection model was used. *G*. *mellonella* infection with *C*. *burnetii* NMII is lethal over a 10-day period and has been used as a model to screen antibiotics [72–74]. Initially AN296 and SF235 were tested for toxicity in *G*. *mellonella* larvae. Each larva was inoculated with 10 μL of PBS containing either Mip inhibitor at various concentrations ranging from 10 μM up to 500 μM, or 1% DMSO as a vehicle control. Larvae were monitored daily, for a period of 10 days and demonstrated no significant toxic effects against the compounds at any of the concentrations tested (S8 Fig). From these results, inhibitor concentrations of 100 μM and 500 μM were selected for efficacy studies. Co-administration of AN296 at 100 μM and 500 μM with *C*. *burnetii* was found to be protective against *C*. *burnetii* induced death as compared to the control (*p* < 0.05, Fig 9A). Curiously, despite being less effective in assays *in vitro*, SF235 at 500 μM with *C*. *burnetii* was also found to be protective against *C*. *burnetii* induced death as compared to the control (*p* < 0.05, Fig 9B). These studies demonstrate that the use of inhibitors against *Cb*Mip is protective against *C*. *burnetii* infection in the more complex *G*. *mellonella* infection model.

## Discussion

The Mip homologue in *C*. *burnetii* (*Cb*Mip) was first described and characterised by Mo *et al*. more than 25 years ago [56]. Mature *Cb*Mip is approximately 24 kDa in size and has a predicted pI of 10.7. Similar to many other Mip proteins, *Cb*Mip consists of two distinct domains, an N-terminal dimerization domain linked by an alpha helix to the C-terminal domain containing the FKBP fold exhibiting PPIase activity which is inhibited by rapamycin [56]. This protein likely forms a homodimer on the outer membrane of the bacterial cell and may also be secreted, at least in axenic media [32,75]. *Cb*Mip is reported to be expressed at comparable levels in both LCVs and SCVs [76] and studies focused on developing putative vaccine candidates or serodiagnostic markers of *C*. *burnetii* infection have identified *Cb*Mip as a highly immunogenic protein in mice and, interestingly, it is seroreactive in Q-fever patients, showing that *Cb*Mip is also expressed *in vivo* [77–80]. However, despite these numerous studies on the *Cb*Mip protein, very little is known about the role of the protein in *C*. *burnetii* pathogenesis.

Previous studies investigating the functional role of Mip, either through mutagenesis or inhibition, in facultative intracellular pathogens such as *L*. *pneumophila* and *B*. *pseudomallei* [35,43,54,81], reported that loss of Mip activity had no impact on growth in the cultivation medium. Therefore, it was initially unexpected that *C*. *burnetii* growth in axenic media was impacted by the presence of inhibitors for *Cb*Mip. However, since *C*. *burnetii* is an obligate intracellular pathogen, to facilitate growth outside of cells, ACCM-2 media has been formulated to mimic the intracellular environment and is unlike the highly permissive growth medium used to culture facultative intracellular pathogens. Using inhibitors against *Cb*Mip we observed a dose dependent effect on *C*. *burnetii* replication in axenic media. These compounds do not merely stop the bacteria from replicating but lead to a loss in bacterial counts suggesting that an outcome of blocking *Cb*Mip activity is a bactericidal effect on *C*. *burnetii*. This observation may also explain why attempts to mutate *cbmip* have not been successful. Attempted targeted mutagenesis was unsuccessful after achieving the merodiploid intermediate; normally the most challenging point to reach using the loop in/loop out protocol for targeted gene inactivation in *C*. *burnetii* [82]. Resolution of the integrated plasmid through sucrose selection only ever yielded wild-type revertants. It cannot be ruled out that this region of the genome is less amenable to homologous recombination, thereby making the generation of a *cbmip* mutant an extremely rare event. However, comparison with an alternative mutagenesis approach which relies on a different molecular mechanism for mutagenesis was done. The *Himar1*-based transposon system developed for *C*. *burnetii* mutagenesis [83] involves the use of a transposable element and accompanying transposase to direct random, nonspecific integration of the transposon (Tn) at T/A base pairs, and has proven to be a successful mutagenesis method for generating mutant libraries in *C*. *burnetii* [21,65,84]. A Tn mutant of *cbmip* was not present in the libraries reported by Newton *et al*. and Martinez *et al*. however it is noted that although the Martinez library generated over 3,000 mutants, only 26.6% of the CDS were mutated. The more recent 10,000 transposon mutant library generated by Metters *et al*. [65] identified two transposon hits in the *cbmip* open reading frame. However, both mutants would permit transcription of a truncated protein with an intact PPIase domain. In this current study, these truncated *Cb*Mip variants were shown to be catalytically active, through recombinant expression. This data is consistent with a study by Mo *et al*. who reported that the *cbmip* transcript contains two internal translation re-initiation sites to facilitate expression of truncated *Cb*Mip analogues with a catalytically active FKBP domain in *C*. *burnetii* [63]. These two internal translation re-initiation sites are also retained in the surviving transposon mutants. These data suggest that the mutants in the Metters *et al*. library retain some *Cb*Mip activity and therefore cannot be considered true *cbmip* mutants. As the truncated *Cb*Mips reported here and earlier all lack a signal sequence, this suggests that the essential role of *Cb*Mip is intracellular and requires FKBP activity. The absence of a catalytically inactive mutant in published *C*. *burnetii* Tn mutant libraries and the inability to generate a *cbmip* mutant using available genetic tools, combined with the findings that high concentrations of small molecule inhibitors against *Cb*Mip are bactericidal against *C*. *burnetii*, strongly indicate that *cbmip* may be an essential gene in *C*. *burnetii*.

Proteomic analysis of *C*. *burnetii* cultures exposed to the inhibitor AN296, at concentrations that inhibited replication but did not reduce bacterial viability counts, revealed that 18 proteins were differentially abundant. One significant change was the increase in four DNA repair proteins, which suggested that the cells were under stress. ACCM-2 media has been reported to represent a high oxidative stress environment [85] and DNA repair is considered part of the oxidative defence mechanisms of *C*. *burnetii* [30]. The increase in DNA repair enzymes led to the hypothesis that the bacterial cells were subject to oxidative stress when incubated with *Cb*Mip inhibitors. This theory was validated using a hydrogen peroxide sensitivity assay where *C*. *burnetii* cells were exposed to a low concentration of H_2_O_2_ [86] in the absence or presence of *Cb*Mip inhibitor at concentrations that still permitted replication. This experiment showed that *C*. *burnetii*, in the presence of *Cb*Mip inhibitors, becomes very sensitive to H_2_O_2_, suggesting that inhibition of *Cb*Mip leads to an increased susceptibility to oxidative stress, resulting in the bacterium responding by upregulating the expression of several DNA repair systems. Professional phagocytes are more efficient at using oxidative burst to kill pathogens [87,88], and as such the proteomics and H_2_O_2_ sensitivity results explain why lower concentrations of *Cb*Mip inhibitors (50 μM), were more effective at inhibiting *C*. *burnetii* replication in THP-1 cells than HeLa cells. This may also be the reason why the inhibitors are also effective at inhibiting *C*. *burnetii* replication at later stages of the infection.

Similar effects of Mip inhibition have been observed in another obligate intracellular pathogen, *C*. *trachomatis*, where exposure to the macrolides, FK506 and rapamycin, or pipecolic acid-based inhibitors, PipN3 and PipN4, resulted in inhibition of intracellular replication and reduced infectivity of *C*. *trachomatis* progeny [54,60]. Even though the Mip protein from *C*. *trachomatis*, *Ct*Mip, has been well studied [54,57,60,89–91], to date no *ctmip* mutant has been described and characterized for *C*. *trachomatis*. Although an axenic culture medium that supports the propagation of *C*. *trachomatis* has not yet been reported, a medium that supports the metabolic activity and survival of *C*. *trachomatis* outside of the host has been developed [92]. It would be interesting to test if inhibitors of *Ct*Mip would have bactericidal activity against *C*. *trachomatis* similar to that observed for *C*. *burnetii*. Such investigation would shed light on whether Mip plays an indispensable role in the lifecycle of obligate intracellular pathogens.

This study demonstrated that AN296 was effective *in vitro* at inhibiting the replication of *C*. *burnetii* post-exposure in THP-1 cells. Addition of AN296 to cells, 2 or 3 days post exposure to *C*. *burnetii*, resulted in a significant decrease in intracellular replication. To further validate AN296 for therapeutic potential, it was screened in the more complex *G*. *mellonella* infection model. This model serves as an excellent starting point for therapeutic studies as the larvae can be maintained at human body temperature (37°C), they have functional homologues to several components of the mammalian innate immune system, and are equally susceptible to both phase I and phase II *C*. *burnetii* strains [74]. In addition, the toxicity and efficacy of several antibiotics tested in the *G*. *mellonella* model have been shown to correlate with the mouse model [93,94] and *G*. *mellonella* larvae also have the capacity to metabolise compounds, another factor that must be taken into consideration when taking new compounds forward as potential therapeutics [95–97]. Importantly the inhibitors were non-toxic to *G*. *mellonella* larvae at the highest dose tested. AN296 was found to protect larvae from infection with *C*. *burnetii* NMII and was effective for at least ten days. Of note is that despite being less effective in assays *in vitro*, SF235 was also found to be protect *G*. *mellonella* against *C*. *burnetii* induced death.

Mip proteins, and FKBPs in general, have been reported to act on several protein targets [47,98,99]. Therefore, it is unsurprising that inhibition of *Cb*Mip activity results in significant changes in the abundance of multiple proteins, which cumulatively can act to reduce pathogen replication. One also cannot at this stage rule out the effect these inhibitors may have on host FKBPs. From the results obtained, SF235 and ANCH37 (AN296) have reduced potency against *Cb*Mip (18±4 μM and 4.6±0.9 μM, respectively) as compared to the values reported against *Bp*Mip to which they were originally developed (0.29+0.06 μM and 0.12 μM, respectively) [55,61]. Despite this, these compounds present excellent starting points for development into more potent and stable molecules for use in further studies including those *in vivo*.

The data presented warrants further investigation into the exact role that *Cb*Mip plays in *C*. *burnetii* biology. In addition, since *Cb*Mip inhibitors SF235 and AN296 were found to also be effective against the virulent phase I strain, these data support the further development of *Cb*Mip inhibitors for increased potency and specificity for *Cb*Mip have potential as novel therapeutics against Q fever.

## Materials and methods

### Reagents

Cell culture media, reagents and fetal bovine serum (FBS) were obtained from Gibco (ThermoFisher Scientific). General chemicals were obtained from Sigma-Aldrich/Merck Millipore unless otherwise stated.

### Culture for bacterial strains and mammalian cell lines

The bacterial strains and plasmids used in this study are shown in S1 Table. *E*. *coli* strains were grown in Luria-Bertani medium and antibiotics were added as necessary to the following final concentrations: ampicillin, 100 μg/mL; kanamycin, 50 μg/mL. Both *C*. *burnetii* NMI (the infectious phase I variant, RSA493), and *C*. *burnetii* NMII (the avirulent phase II variant, RSA439, clone 4) were used in the growth experiments in axenic culture, and *C*. *burnetii* NMII was used for mutagenesis, cell infection assays and the *G*. *mellonella* infection model.

*C*. *burnetii* was routinely grown in acidified citrate cysteine medium-2 (ACCM-2) media from Sunrise Science Products (San Diego, CA) at 37°C in 5% CO_2_ and 2.5% O_2_ [100] to stationary phase (6–7 days) before harvesting for infection or growth assays. When required chloramphenicol and kanamycin were used in *C*. *burnetii* ACCM-2 cultures at 3 μg/mL and 350 μg/mL, respectively. ACCM-2 agarose plates were prepared using UltraPure Agarose (Invitrogen) and additionally supplemented with 0.50 mM L-tryptophan [101].

All manipulations of *C*. *burnetii* NMI were carried out in a class III microbiological safety cabinet complying with British Standard EN12469:2000 and all studies were risk assessed and approved by Dstl’s Biosafety Committee.

THP-1 human monocytic cells and HeLa human carcinoma cells were maintained in RPMI 1640 supplemented with GlutaMAX and 10% (v/v) heat-inactivated FBS at 37°C in 5% CO_2_ unless otherwise described.

### Quantification of *C*. *burnetii* genome equivalents

For studies using *C*. *burnetii* NMII, genome equivalents (GE) of *C*. *burnetii* were quantified using the Quant-iT PicoGreen dsDNA assay kit (ThermoFisher Scientific) or by quantitative PCR (qPCR), using *ompA* specific primers, as previously described [102,103]. PicoGreen assays were performed following the manufacturers protocol and results were read using the POLARStar plate reader (BMG Labtech), data were processed using the MARS analysis software (BMG Labtech) and analyzed using Microsoft Excel. Quantification by qPCR was performed using a QuantStudio 3 real-time PCR system (Applied Biosystems, Thermo Fisher Scientific). QuantStudio Design and Analysis Software was used to generate standard curves and perform initial analysis. Data was exported to Microsoft Excel for further analysis. For studies using *C*. *burnetii* NMI, the CFU/mL of single use frozen *C*. *burnetii* stocks was quantified by plating out in serial dilutions on ACCM-2 agar plates which were then left for 7–10 days before viability counts were performed.

### Growth assays in axenic media

#### *Studies involving Phase I strain*, *C*. *burnetii* NMI

*Growth curves*. Vented flasks containing 5 mL of ACCM-2 media supplemented with 0.50 mM L-tryptophan and containing either 100 μM of SF235 or AN296 or DMSO vehicle control were inoculated at 1 × 10^4^ CFU/mL using freshly thawed stocks of *C*. *burnetii* NMI. Cultures were incubated statically at 37°C in a Galaxy 170 R incubator (New Brunswick Scientific) adjusted to 5 % CO_2_ and 2.5 % O_2_. Samples of 100 μL were removed at days 0, 1, 2, 3, 4 and 7 and plated in serial dilutions on ACCM-2 agar plates which were then left for 7–10 days before viability counts were performed. For delayed dosing experiments, the assay was prepared without inhibitors as described above and on day 3 of the growth curve, 25 μL of inhibitor or DMSO diluted in media was added to each culture.

#### *Studies involving Phase II strains*, *C*. *burnetii* NMII

*Luminescence assays*. Stationary phase (6–7 day) ACCM-2 cultures of the luciferase-expressing *C*. *burnetii* NMII strain, *C*. *burnetii*-lux, were quantified using the PicoGreen assay and appropriately diluted to 1 × 10^6^ genome equivalents (GE)/mL in white 96-well plates (Corning) in 0.1 mL of fresh ACCM-2 medium per well, in the presence of varying concentrations of Mip inhibitor. Bioluminescence was measured every 24 h over 5 days by a POLARStar plate reader (BMG Labtech). For delayed dosing experiments, the assay was prepared without inhibitors as described above and 25 μL of inhibitor or DMSO diluted in ACCM-2 was added to each well on day 2 or day 3 of the assay.

*CFU assay*. Using *C*. *burnetii* NMII and clear 96-well plates (Corning), the growth assay was prepared as described above for the Luminescence assay. Following 4 days of incubation, samples from test wells were taken and plated in serial dilutions on ACCM-2 agar plates which were then left for 7 days before viability counts were performed.

#### Sensitivity to hydrogen peroxide assays

Stationary phase (6–7 day) ACCM-2 cultures of *C*. *burnetii* NMII were quantified using the PicoGreen assay and appropriately diluted to 1.0 × 10^6^ genome equivalents (GE)/mL in clear 96-well plates (Corning) in 0.1 mL of fresh ACCM-2 medium containing either 50 μM AN296 or DMSO vehicle control, in the absence or presence of 100 μM H_2_O_2_. The plate was incubated for 4 days before the cultures were plated out to determine viable CFU/mL.

### MIC and MBC assays

#### *Studies involving Phase I strain*, *C*. *burnetii* NMI

Vented flasks containing 5 mL of ACCM-2 media supplemented with 0.50 mM L-tryptophan and containing increasing concentrations of Mip inhibitor were inoculated at 1 × 10^5^ CFU/mL using freshly thawed stocks of *C*. *burnetii* NMI. Cultures were incubated statically at 37°C for 6 days. The MIC was determined both by measuring the OD and then by plating out each broth to obtain a viable bacterial count. The MBC was determined by plating out neat broth from flasks with no visible growth onto ACCM-2 plates.

#### *Studies involving Phase II strains*, *C*. *burnetii* NMII

Stationary phase (6–7 day) ACCM-2 cultures of *C*. *burnetii* NMII were quantified using the PicoGreen assay and appropriately diluted to 2.5 × 10^5^ genome equivalents (GE)/mL in clear 24-well plates (Nunc) in 1 mL of fresh ACCM-2 medium supplemented with 0.50 mM L-tryptophan per well, in the presence of increasing concentrations of Mip inhibitor, and then incubated for 6 days. The MIC was determined both by measuring the OD and then by plating out each broth to obtain a viable bacterial count. The MBC was determined by plating out neat broth of wells with no visible growth onto ACCM-2 plates.

### Mutagenesis of *cbmip (*CBU0630)

Mutagenesis of *cbmip* (*cbu0630*) was attempted in *C*. *burnetii* NMII following the loop in/loop out method for targeted gene inactivation described by Beare *et al*. [82]. All oligonucleotides used in this study are shown in S2 Table. The 2-kb fragments of the 5′- and 3′-end-flanking regions of *cbmip* were first amplified from genomic DNA by PCR using the upstream and downstream oligonucleotide pairs cbu0630-up F and cbu0630-up R, and cbu0630-down F and cbu0630-down R, respectively. These two PCR fragments were purified and joined together by strand overlapping extension (SOE) PCR [104], using primers cbu0630-up F and cbu0630-down R, to generate a PCR product containing a unique internal *Not*I site between the 5′- and 3′- flanking regions and flanked by *Bam*HI and *Sal*I restriction sites. This 4-kb fragment was cloned into *Bam*HI/*Sal*I-digested pJC-CAT, generating plasmid pJC-CAT::cbu0630-prep. The *1169*^*P*^*-Kan* cassette was then amplified from pJB-Kan by PCR using oligonucleotides P1169-Kan-NotI F and P1169-Kan-NotI R, treated with *Not*I and cloned into *Not*I-digested pJC-CAT::cbu0630-prep to create the deletion plasmid pJC-CAT::cbu0630-Kan. This construct was electroporated into *C*. *burnetii* NMII and chloramphenicol and kanamycin resistant colonies were successfully isolated and confirmed as *cbu0630* integrants through PCR analysis of isolated genomic DNA. Following sucrose selection, a limited number of very small colonies were isolated however they either failed to grow or were confirmed as wild-type revertants.

### Cloning and expression of *Cb*Mip

The nucleotide sequence encoding *C*. *burnetii mip* without the predicted N-terminal signal peptide (nucleotides 64 to 690, corresponding to amino acids 22–230) was synthesized with codon optimization for expression in *E*. *coli* by GenScript (Piscataway, NJ) and was received as plasmid pMK-cbMipOpt. The *cbmip* coding sequence was amplified by PCR from pMK-cbMipOpt as a template using primers CbMip_NcoI F and CbMip_BamHI R to introduce flanking *Nco*I and *Bam*HI sites. The amplified DNA fragment was first subcloned into pCR-Blunt II-TOPO and then excised by *Nco*I and *Bam*HI restriction digest and cloned into the same restriction sites of the expression vector pETM-11 (European Molecular Biology Laboratory, [105]), which encodes an N-terminal hexahistidine tag followed by a TEV site, to generate pETM-11-cbMip.

Truncated *cbmip* gene constructs corresponding to transposon mutants, *cbmip*-TM1 (nucleotides 102 to 690, corresponding to amino acids 36–230) and *cbmip*-TM2 (nucleotides 213 to 690, corresponding to amino acids 71–230), were generated following the same methodology described above for the full length *cbmip* using the appropriate primer pairs for each truncated construct. After transformation into chemically competent *E*. *coli* TOP10, positive clones were verified by DNA sequencing (AGRF, Australia, or Macrogen, South Korea). The codon optimized nucleotide sequence of *cbmip* and the protein sequences for each construct are listed in S3 Table.

### Protein production and purification

Recombinant plasmids were transformed into the *E*. *coli* strain BL21(DE3)pLysS and grown with shaking in 1 L LB medium supplemented with 50 μg/mL kanamycin at 37°C to an optical density at 600 nm of 0.4, whereupon protein production was induced for 2 h by the addition of 1.0 mM IPTG. Cells were cooled on ice for 15 min and harvested by centrifugation at 3,000 x *g* for 15 min at 4°C, and cell pellets were stored at -20°C until further processing. The cell pellets were resuspended in 10 mL/g Buffer A (50 mM HEPES, 150 mM NaCl, 10% (v/v) glycerol, 25 mM imidazole, pH 7.5) with the addition of lysozyme (0.2 mg/mL, Sigma-Aldrich), complete EDTA-free protease inhibitor cocktail (Roche) and deoxyribonuclease (DNAse I, 1 μg/mL, Roche), lysed using an EmulsiFlex C5 homogeniser (Avestin) and clarified at 24,000 x *g* for 30 min at 4°C. The soluble supernatant was passed through a 0.22 μm filter and then applied onto a 5 mL HisTrap HP column (Cytiva) that had been pre-equilibrated with Buffer A using an ÄKTA Start (Cytiva). After washing with Buffer A, the 6xHis tagged protein was eluted using a gradient of 1–100% Buffer B (50 mM HEPES, 150 mM NaCl, 10% (v/v) glycerol, 500 mM imidazole, pH 7.5). Fractions containing the protein of interest were pooled, concentrated using centrifugal filter (Amicon Ultra, MWCO 10 kDa). This was followed by size-exclusion chromatography on a HiLoad 16/600 Superdex 75pg (GE Healthcare Life Sciences) column in 50 mM HEPES, 150 mM NaCl, 10% (v/v) glycerol, pH 7.5. Again, fractions containing the protein of interest were pooled and concentrated using centrifugal filter. Proteins were assessed as having >95% purity by SDS–PAGE. The concentration was calculated from the A_280_ and the theoretical extinction coefficient calculated using the ProtParam tool from ExPASy. Protein aliquots were snap frozen in liquid nitrogen and stored at -80°C.

### Enzymatic assays

Purified recombinant *Cb*Mip proteins were tested for PPIase activity in an enzyme assay by measuring the *cis*-*trans* isomerization of the tetrapeptide Suc-Ala-Phe-Pro-Phe-*p*-nitroanilide (Bachem #4016001). The peptidyl-prolyl *cis*-*trans* isomerase assays were performed using a protease-coupled assay as previously described [55,62]. Data were determined for enzyme at 0, 6.25, 12.5, 25, 50, 75, 100, 150, 200, 300, 450, 600 nM, with three replicates. Data were analysed as previously described [55]. Briefly, the *k*_*obs*_ was calculated using one-phase association, upon which the *k*_*enz*_ was determined using the equation *k*_*enz*_ = *k*_*obs*_*—k*_*uncatalysed*_. The specificity constant, *k*_*cat*_*/K*_*M*_, was fit to the equation; *k*_*cat*_*/K*_*M*_ = *k*_*enz*_/[PPIase] using linear regression in GraphPad Prism v. 9.0.1. The inhibition constant (*K*_i_) of SF235 and ANCH37 against *Cb*Mip were determined using a revised Morrison equation as previously described [55]. All enzymatic data were fitted using Graphpad Prism v. 9.0.1.

### Protein modeling with AlphaFold2

Protein models were made with AlphaFold v.2.2.0 [66], running on a local server using the 2022-03-03 database. Models were made in multimeric mode with an input of two copies of the full length *Cb*Mip with the signal sequence removed, *Cb*Mip-TM1, or *Cb*Mip-TM2. Five models were made for each input sequence, superimposed using PyMOL v.2.5.3 (Schrödinger). Models were manually inspected, and figure prepared, using PyMOL.

### *C*. *burnetii* intracellular growth assays

Growth assays in THP-1 and HeLa cells were performed similarly to those described previously [103]. Briefly, THP-1 cells were seeded at 5 × 10^5^ into 24-well flat-bottom tissue culture plates (Nunc) and differentiated with 10 nM phorbol 12-myristate 13-acetate (PMA; Sigma-Aldrich) for 3 days. HeLa cells were seeded at 5 × 10^4^ cells/well and incubated for 24 h prior to infection. Samples for immunofluorescence (IF) were seeded onto 13-mm sterile glass coverslips. *C*. *burnetii* NMII cultures were quantified as described above and diluted appropriately into RPMI 1640 supplemented with 5% (v/v) FBS to infect THP-1 cells at a multiplicity of infection (MOI) of 5 and HeLa cells at a MOI of 50. Diluted *C*. *burnetii* NMII were pre-treated for 1 h at 37°C with defined concentrations of Mip inhibitor or DMSO vehicle control (final concentration of DMSO was 0.1%) and subsequently 0.5 mL aliquots per well were used to infect cells. Upon infection, cells were centrifuged at 500 x *g* for 5 min and then incubated for 4 h at 37°C and 5% CO_2_ before being washed once in PBS to remove extracellular bacteria and supplemented with fresh medium containing Mip inhibitor or DMSO as control. At indicted time points, cells were either fixed for microscopy or lysed with H_2_O and collected for quantification of *C*. *burnetii* intracellular replication. For day 1, 3, 5 and 7 day post infection samples, media from each duplicate well was pooled and collected alongside the lysed cells. Following lysis, samples were centrifuged at 17,000 x *g* for 20 min at 4°C before gDNA was extracted from the resulting pellet using the Quick-DNA Miniprep kit/Zymo gDNA extraction kit (Zymo Research) and quantified by qPCR.

### Immunofluorescence microscopy

At indicated time points, infected cells were fixed for 20 min at room temperature with 4% (w/v) paraformaldehyde (in PBS). After washing in PBS, samples were first blocked in PBS containing 5% (v/v) FBS and 0.05% (w/v) saponin (Sigma-Aldrich) (blocking buffer). Samples were then stained using primary antibodies diluted in blocking buffer: 1:50 anti-LAMP-1 clone H4A3, supernatant (H4A3 was deposited to the DSHB by August, J.T. / Hildreth, J.E.K. (DSHB Hybridoma Product H4A3)) and 1:10,000 rabbit anti-*C*. *burnetii* antibodies (Roy Laboratory, Yale University). Secondary antibodies, anti-mouse Alexa Fluor 488 and anti-rabbit Alexa Fluor 568 (Thermo Fisher Scientific) were used at a dilution of 1:3,000 in blocking buffer. DNA was stained using Hoechst 33258 (1:10,000; Thermo Fisher Scientific) and coverslips were mounted onto glass slides using ProLong Gold Antifade Mountant (Thermo Fisher Scientific). Fluorescence microscopy was performed using a Nikon A1Si confocal microscope, and images were acquired using NIS-Elements software (Nikon).

### Inhibitor cytotoxicity assays

Cytotoxicity induced by SF235 and AN296 over the time period of a *C*. *burnetii* intracellular growth assay was assessed in both HeLa and THP-1 cells.

#### IC_50_ determination

The cytotoxicity of the inhibitors themselves, in the absence of *C*. *burnetii* infection, was investigated by determining their IC_50_ values at several time points, for up to 6 days of incubation. Cell viability was determined colorimetrically using the Cell proliferation reagent WST-1 (Roche, Basel, Switzerland). Cells were seeded in triplicate in 96-well plates. HeLa cells were prepared as a stock solution of 1 × 10^4^ cells/mL, wells were then seeded with 100 μL of diluted stock solution as appropriate for the incubation time point (t_1_ = 100%; t_2_ = 50%; t_3_ = 30%; t_4_ = 25% and t_5_ = 20%). Similarly, THP-1 cells were prepared as a stock solution of 5 × 10^5^ cells/mL. The two inhibitors were diluted according to their individual water solubility in cell media to give a maximum concentration of 400 μM for SF235 and 200 μM for AN296 and 2% DMSO. This working concentration was serially diluted 1:1 in media and a 100 μL aliquot of each inhibitor concentration was added to the cells, resulting in the final inhibitor concentrations ranging from 0.391 μM to 200 μM for SF235 and 0.391 μM to 100 μM for AN296. The final concentration of DMSO was 1% at the highest concentration of inhibitor tested and less at each of the dilutions. A dilution series of DMSO (starting at 1%) served as a control for each cell line at each assessed time point. Untreated cells were used as controls. The cells were incubated at 37°C and 5% CO_2_ for the indicated period of time. To determine cell viability, 10 μL of WST-1 was added to each well according to the manufacturer’s instructions. After 1 h (HeLa cells) or 4 h (THP-1 cells) of incubation, the absorbance of the soluble formazan product at 450 nm and the background at 630 nm were determined using a Tecan infinite 200Pro microplate reader (Tecan Trading AG, Männedorf, Switzerland). Data were analysed and IC_50_ values were determined using GraphPad Prism v9.01.

#### LDH release assay

The inhibitor concentrations tested were the same as those used in the *C*. *burnetii* intracellular growth assays in THP-1 cells (50 μM) and the control was 0.1% DMSO. LDH release was measured after incubating cells with inhibitors for 4 h (representing the day 0 infection time point), 28 h (day 1), 76 h (day 3) and 124 h (day 5) using the Roche LDH Cell Cytotoxicity kit (#11644793001), following the manufacturer’s instructions. At each indicated time point, a positive control (Triton X-100) and negative control (media only) were included in in triplicate. All results are presented as the mean of three independent experiments containing two technical repeats. Cytotoxicity was calculated as the percentage of lactate dehydrogenase (LDH) measured in Triton X-100 for that assay and are represented relative to the control (0.1% DMSO).

### Infection of *Galleria mellonella* larvae

*G*. *mellonella* were maintained and infected as previously reported [74,103]. Larvae were grown in-house and kept at 30°C in the dark until use. To test the toxicity of the inhibitors, 50 mM stock solutions of compound dissolved in DMSO were diluted in PBS to the desired concentrations and the final concentration of DMSO was kept at 1% (v/v). A 10 μL aliquot was injected into the right proleg of *G*. *mellonella* larvae, which were then kept isolated at 37°C in the dark. Survival was assessed every 24 h for 10 days. To assess the effect of Mip inhibitors on *C*. *burnetii* pathogenicity, 10^8^ GE/mL of *C*. *burnetii* NMII was incubated in the presence of 0, 100 μM or 500 μM of compound in PBS with a final concentration of 1% DMSO, at 37°C for 1 h. A 10 μL aliquot of this mixture was then injected into larvae and survival tracked for 10 days as above. All treatment groups started with 12 larvae. Kaplan-Meier survival curves were determined from three independent experiments. Data was analysed using the log-rank (Mantel-Cox) test with Bonferroni correction for multiple comparisons with a significance level of 0.05.

### Proteomic analysis

Stationary phase ACCM-2 cultures of *C*. *burnetii* NMII grown up directly from glycerol stocks were quantified using the PicoGreen assay and appropriately diluted to 1 × 10^6^ genome equivalents (GE)/mL into 20 mL of fresh ACCM-2. Cultures were incubated for 72 h prior to the addition of 100 μM AN296 or vehicle control. Final DMSO concentration was kept at 0.2% (v/v) for all cultures. After 24 h, cultures were harvested by centrifugation and washed twice with PBS. Total protein was extracted from *C*. *burnetii* cell pellets using chloroform and methanol, following previously published procedures [106]. Total protein content was assessed using a bicinchoninic acid protein assay (Pierce, Thermo Fisher Scientific) according to the manufacturer’s instructions.

#### Protein clean-up and in-solution digestion

Quantified precipitated samples were resuspended in 100 μL of 5% SDS by boiling for 10 min at 95°C. Samples were then reduced with 10 mM DTT for 10 min at 95°C and then alkylated with 40 mM chloroacetamide for 1 h in the dark. Reduced/alkylated samples were then cleaned up using Micro S-traps (https://protifi.com/pages/s-trap) according to the manufacturer’s instructions. Samples were then digested for 4 h with trypsin/lys-c (~1:25 protease/protein ratio), collected and dried. Dried samples were then further cleaned up with home-made high-capacity StageTips composed of 1 mg Empore C18 material (3 M) and 5 mg of OLIGO R3 reverse phase resin (Thermo Fisher Scientific) as described [107,108]. Columns were wet with Buffer B (0.1% formic acid, 80% acetonitrile) and conditioned with Buffer A* (0.1% TFA, 2% acetonitrile) prior to use. Acidified samples were loaded onto conditioned columns, washed with 10 bed volumes of Buffer A* and bound peptides were eluted with Buffer B before being dried then stored at -20°C.

#### LFQ-based quantitative proteome liquid chromatography-mass spectrometry

Dried proteome digests were re-suspended in Buffer A* and separated using a two-column chromatography set up composed of a PepMap100 C18 20 mm x 75 μm trap and a PepMap C18 500 mm x 75 μm analytical column (Thermo Fisher Scientific). Samples were concentrated onto the trap column at 5 μL/min for 5 min with Buffer A (0.1% formic acid, 2% DMSO) and then infused into an Orbitrap Q-Exactive plus Mass Spectrometer (Thermo Fisher Scientific) at 300 nL/min via the analytical column using a Dionex Ultimate 3000 UPLC (Thermo Fisher Scientific). 135-min analytical runs were undertaken by altering the buffer composition from 2% Buffer B (0.1% formic acid, 77.9% acetonitrile, 2% DMSO) to 22% B over 105 min, then from 22% B to 40% B over 10 min, then from 40% B to 80% B over 5 min. The composition was held at 80% B for 5 min, and then dropped to 2% B over 2 min before being held at 2% B for another 8 min. The Q-Exactive Mass Spectrometer was operated in a data-dependent mode automatically switching between the acquisition of a single Orbitrap MS scan (375–1400 m/z, maximal injection time of 50 ms, an Automated Gain Control (AGC) set to a maximum of 3 × 10^6^ ions and a resolution of 70k) and 15 Orbitrap MS/MS HCD scans (stepped NCE of 28;30;34, a maximal injection time of 65 ms, an AGC set to a maximum of 1 × 10^5^ ions and a resolution of 17.5k).

#### Mass spectrometry data analysis

Proteome datasets were processed using MaxQuant (v1.6.17.0.) [109] and searched against the *C*. *burnetii* strain RSA 493 / Nine Mile phase I and Dugway 5J108-111 proteomes (Uniprot accession: UP000002671 and UP000008555, respectively). Both the Dugway 5J108-111 and RSA 493 / Nine Mile phase I proteomes were included to enable the mapping / incorporation of Uniprot assignment from both strains. Searches were undertaken using “Trypsin” enzyme specificity with carbamidomethylation of cysteine as a fixed modification. Oxidation of methionine and acetylation of protein N-termini were included as variable modifications and a maximum of 2 missed cleavages allowed. To enhance the identification of peptides between samples, the Match between Runs option was enabled with a precursor match window set to 2 min and an alignment window of 20 min with the label free quantitation (LFQ) option enabled [110]. The resulting outputs were processed within the Perseus (v1.6.0.7) analysis environment [111] to remove reverse matches and common protein contaminates prior to further analysis. For LFQ comparisons biological replicates were grouped and missing values were then imputed based on the observed total peptide intensities with a range of 0.3σ and a downshift of 2.5σ using Perseus. Student t-tests were undertaken to compare the proteomes between groups. The resulting MS data and search results have been deposited into the PRIDE ProteomeXchange Consortium repository [112,113] with the dataset identifier PXD036679.

### Synthesis of pipecolic acid based Mip inhibitors

Inhibitors SF235, ANCH37 and AN296 were prepared as previously described [50,61] (Australian Patent Application No. PCT/AU2023/050201).

### Stability assessment of AN296 at pH = 4.75

For this purpose, Buffer C (S4 Table) was prepared which mimicked the inorganic components of ACCM-2 media and was adjusted to pH 4.75 using citrate buffer. A 100 μM stock solution of AN296 was then prepared in Buffer C and incubated at 37°C for a period of 7 days, corresponding to cell assay conditions with sampling performed daily. Analysis of 50 μL aliquots using HPLC involved an isocratic method with a mobile phase composition of MilliQ/ACN (45%/55% (v/v)) with a flow rate of 1 mL/min and UV-metric detection at 250 nm and 260 nm. A Knauer Eurospher II 100–5 C18 H (150x4.6 mm) (KNAUER Wissenschaftliche Geräte GmbH, Berlin, Deutschland) column was used as the stationary phase. Daily measurements were performed on 3 samples with 2 injections each, as well as AN296 in methanol for retention time control. The first analysis was performed immediately after dilution and before incubation and thus served as a reference point for calculating the content.

#### Chemicals and reference substances

All reagents were of analytical grade. HPLC grade acetonitrile from VWR International GmbH (Darmstadt, Germany). Water for HPLC was purified using a Milli-Q purification system by Merck Millipore (Schwalbach, Germany).

#### Apparatus

HPLC experiments were performed on an Agilent 1100 modular chromatographic system (Agilent technologies, Waldbronn, Germany) consisting of a vacuum degasser (G1322A), a binary pump (G1312A), an autosampler (G1313A), a thermostated column compartment (G1316A) and a diode array detector (G1315B). Agilent ChemStation Rev C 01.10 software was used for data processing. For incubation a Grant Boekel HIS25 Incubator (Grant Instruments, Cambridge, England) was used.

### Statistical analysis

All numerical results were analysed using Microsoft Excel 2010. Statistical analyses were performed using GraphPad Prism, v. 9.0.1.

## Supporting information

S1 Fig Conserved nature of characterized Mip proteins from Gram-negative intracellular pathogens. (A) Multiple sequence alignment of Mip proteins from *C*. *trachomatis* (*Ct*Mip), *B*. *pseudomallei* (*Bp*Mip) *C*. *burnetii* (*Cb*Mip) and *L*. *pneumophila* (*Lp*Mip) using CLUSTAL O (1.2.4). The start of the highly conserved catalytic/PPIase domain is indicated by an open triangle. The essential amino acid (Asp) in *Lp*Mip for PPIase activity is conserved in all proteins and is indicated by a closed triangle. (B) Percentage identity matrix of the PPIase domain of Mip from *C*. *trachomatis* (*Ct*Mip-cat), *B*. *pseudomallei* (*Bp*Mip-cat) *C*. *burnetii* (*Cb*Mip-cat) and *L*. *pneumophila* (*Lp*Mip-cat) using CLUSTAL 2.1.
(TIF)

S2 Fig SF235 and ANCH37 inhibition of *Cb*Mip. Data were collected in a single experiment for *Cb*Mip as described in the experimental section. Each inhibitor concentration of SF235 (closed circle) and ANCH37 (blue square) was tested three times. Data were fitted to equation as described previously [55]. Results are representative of at least two experiments conducted on separate days with different preparations of inhibitor. Error bars show standard error of the mean.
(TIF)

S3 Fig Addition of AN296 after the infection period is also effective at inhibiting intracellular replication of *C. burnetii*. Intracellular replication of *C*. *burnetii* NMII in THP-1 cells in the presence of 50 μM AN296 (blue triangle), 31 μM chloramphenicol (Cm) (open circle) or control (closed circle), introduced after the 4 h infection period. Error bars represent standard error of the mean (*n* ═ 5). ****, *p* < 0.0001. *p* values were determined using two-way ANOVA, followed by Dunnett’s multiple comparison post-test.
(TIF)

S4 Fig Inhibitor-induced cytotoxicity. (A) IC_50_ values for SF235 and AN296 and DMSO after incubation for the indicated periods of time were determined in THP-1 and HeLa cells using the Cell Proliferation Reagent WST-1. (B) Cytotoxicity was measured after THP-1 cells were incubated with SF235 (50 μM), AN296 (50 μM), chloramphenicol (cm, 31 μM) or control (0.1% DMSO) for the indicated period of time using Roche LDH Cell Cytotoxicity kit. No significant difference in cytotoxicity was found between the inhibitors and the control (0.1% DMSO). Data is presented as percent cytotoxicity relative to the control with error bars representing the SD from three independent experiments; *p* values were determined using two-way ANOVA followed by Dunnett’s multiple comparison post-test.
(TIF)

S5 Fig Transposon insertion mutants of the *mip* locus indentified in *C. burnetii* NMII. Overview of transposon mutants generated in the *Coxiella burnetii* NMII Metters *et al*. library. Confirmed transposon insertion sites are indicated by red vertical lines. All TA insertion sites, possible locations where the transposable element can intergrate, are indicated by black vertical lines. (A) Transposon insertion sites around the *cbmip* (*cbu0630*) genetic locus. (B) Enhanced view of transposon insertion sites within the *cbmip* gene. Two out of a possible 37 mutants were identified in the transposon mutant library. (C) Detailed analysis of transposon insertion sites in *cbmip*. In lower case text is the DNA coding sequence for the *C*. *burnetii mip* gene (*cbu0630*) of strain *C*. *burnetii* NMII. Below, in upper case text, is the encoded amino acid sequence, green box indicates the PPIase catalytic domain. The two transposon insertion sites (TA) identified in the transposon mutant library are indicated by blue triangles. The first ATG following the transposon site is indicated by red boxes. In both instances of transposon insertion in the *cbmip* sequence, the downstream ATG from which protein translation would initiate is in-frame with the original full-length gene and therefore would result in the production of a truncated *Cb*Mip protein in the *C*. *burnetii cbu0630* transposon mutants.
(TIF)

S6 Fig Stability profile of AN296 under axenic assay conditions. The stability of compound AN296 under acidic conditions (pH of 4.75), was investigated by HPLC chromatography. A stock solution of AN296 (100 μM) was prepared in Buffer C (composition in S4 Table) which mimicked the inorganic components of ACCM-2 media and was incubated at 37°C for a period of 7 days. Data is presented as the amount of AN296 remaining compared to day 0 (at 250 nm and 260 nm), with error bars representing the standard deviation.
(TIF)

S7 Fig *Cb*Mip inhibition reduces *C. burnetii* replication in axenic media in a dose dependant manner. Bioluminescence was measured as an indicator of *C*. *burnetii*-lux replication. The strain was inoculated at a concentration of 1 × 10^6^ GE/mL into ACCM-2 media with (A) 50 μM or (B) 25 μM of *Cb*Mip inhibitors SF235 (grey square), AN296 (closed triangle) or vehicle control (open circle) and grown over 5 days. Data is presented as RLU (relative light units) with error bars representing the standard deviation from three independent experiments. **, *p* < 0.01; ***, *p* < 0.001; ****, *p* < 0.0001. *p* values were determined using two-way ANOVA, followed by Dunnett’s multiple comparison post-test.
(TIF)

S8 Fig *Cb*Mip inhibitors SF235 and AN296 are non-toxic to *G. mellonella* larvae. Each larva received a single 10 μL injection of in PBS containing either (A) AN296 or (B) SF235 or vehicle control into the right proleg. Inhibitors were tested at increasing concentrations starting at 10 μM and increasing up to 50 μM, 250 μM and 500 μM (groups of *n* ═ 10). Larvae were monitored everyday via twitching response over 10 days. All but two larvae, which received 10 μM of AN296, survived the entire duration of the experiment.
(TIF)

S1 Table List of strains and plasmids used in this study. (DOC)

S2 Table List of oligonucleotides used in this study. (DOC)

S3 Table The codon optimized sequence of *cbmip* and the expected protein sequences of *Cb*Mip and the truncates, *Cb*Mip-TM1 and *Cb*Mip-TM2, for recombinant protein expression. (DOC)

S4 Table Buffer C composition. (DOC)

S1 Data Excel spreadsheet containing, in separate sheets, the underlying numerical data and statistical analysis for Figure panels 2A, 2B, 3C, 4A, 4B, 4C, 5A, 5B, 5C, 5D 5E, 6, 7A, 7B, 8A, 8B, 9A, 9B, S2, S3, S4A, S4B, S6, S7A, S7B, S8A, and S8B. (XLSX)
