# Supplementary figures and images for: Macrophage infectivity potentiator protein, a peptidyl prolyl cis-trans isomerase, essential for Coxiella burnetii growth and pathogenesis
Source: PLoS Pathog. 2023 Jul 3;19(7):e1011491. doi: 10.1371/journal.ppat.1011491 (PMC10348545; doi:10.1371/journal.ppat.1011491)

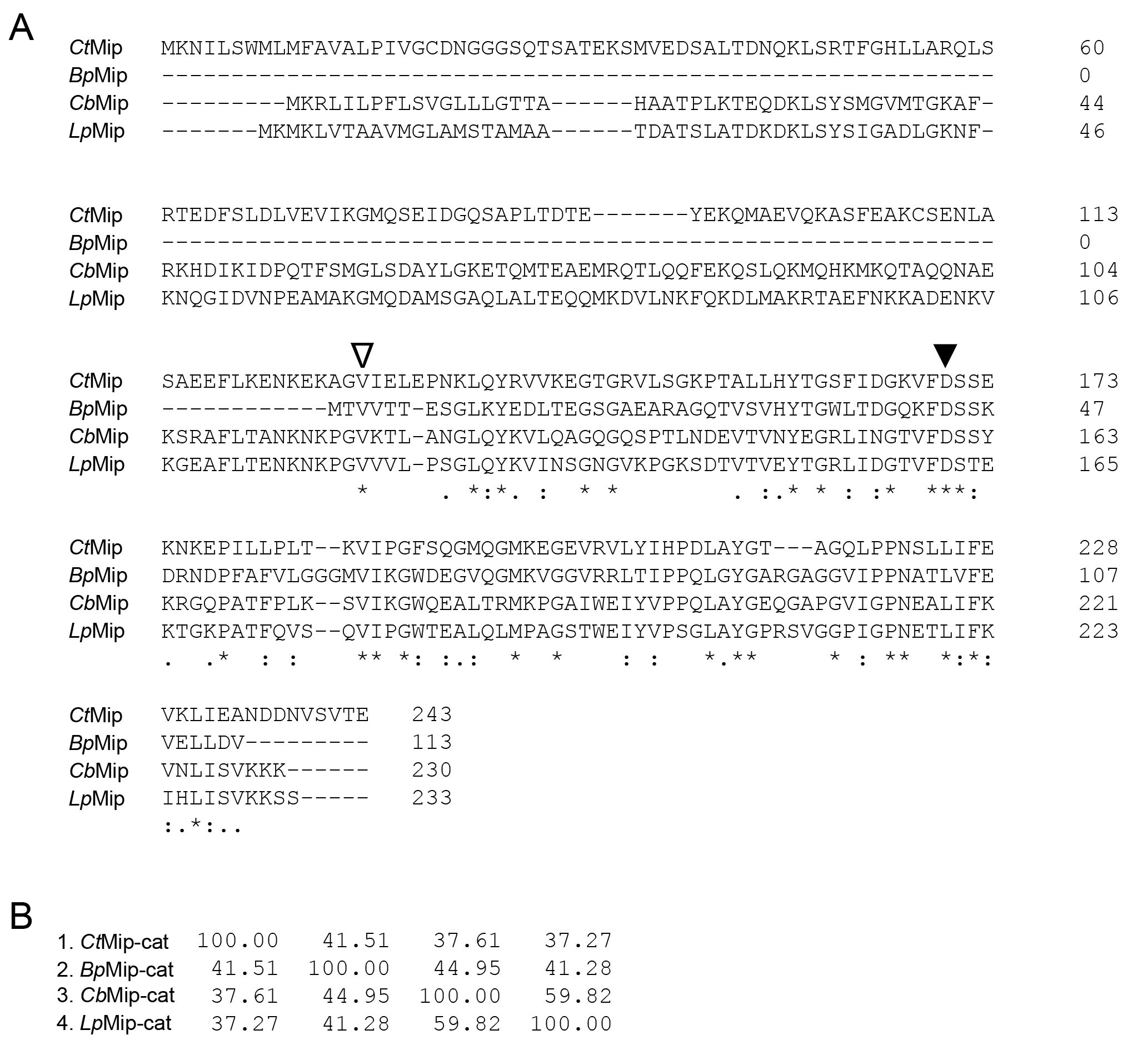

Supplement: S1 Fig — (A) Multiple sequence alignment of Mip proteins from C. trachomatis (CtMip), B. pseudomallei (BpMip) C. burnetii (CbMip) and L. pneumophila (LpMip) using CLUSTAL O (1.2.4). The start of the highly conserved catalytic/PPIase domain is indicated by an open triangle. The essential amino acid (Asp) in LpMip for PPIase activity is conserved in all proteins and is indicated by a closed triangle. (B) Percentage identity matrix of the PPIase domain of Mip from C. trachomatis (CtMip-cat), B. pseudomallei (BpMip-cat) C. burnetii (CbMip-cat) and L. pneumophila (LpMip-cat) using CLUSTAL 2.1. (TIF) [file ppat.1011491.s001.tif]

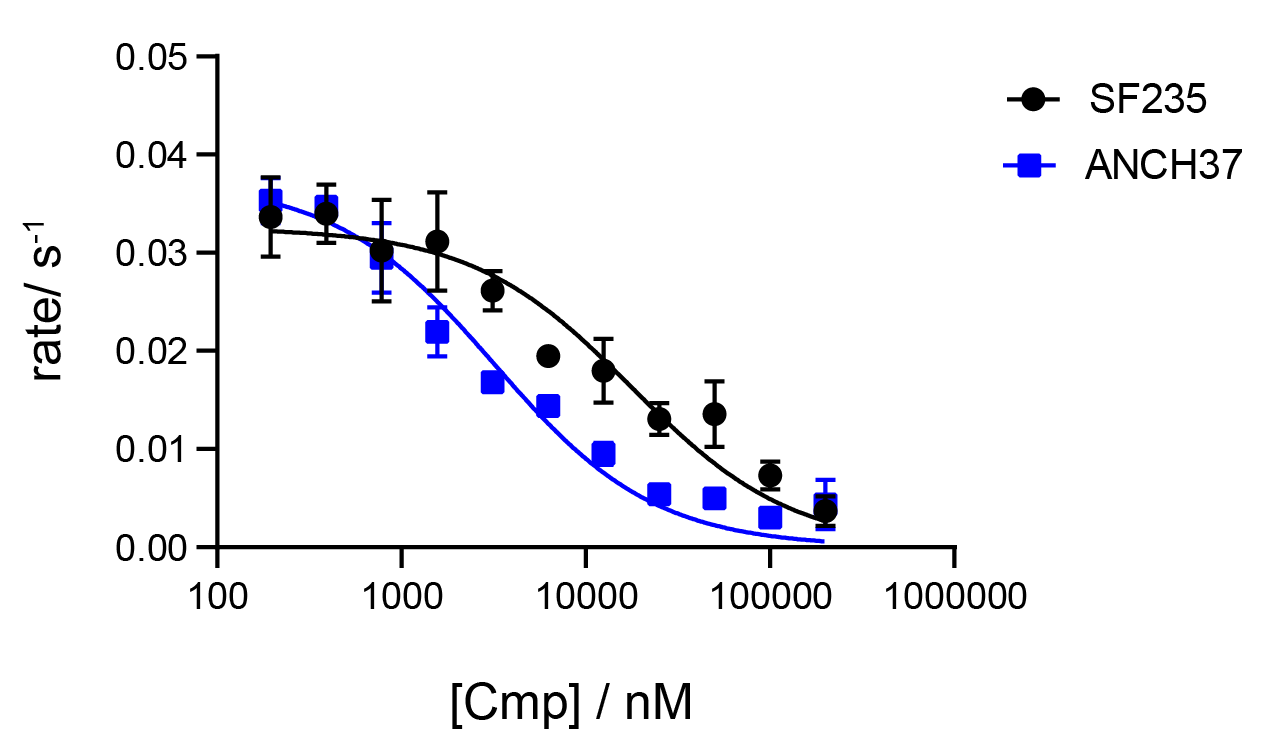

Supplement: S2 Fig — Data were collected in a single experiment for CbMip as described in the experimental section. Each inhibitor concentration of SF235 (closed circle) and ANCH37 (blue square) was tested three times. Data were fitted to equation as described previously [55]. Results are representative of at least two experiments conducted on separate days with different preparations of inhibitor. Error bars show standard error of the mean. (TIF) [file ppat.1011491.s002.tif]

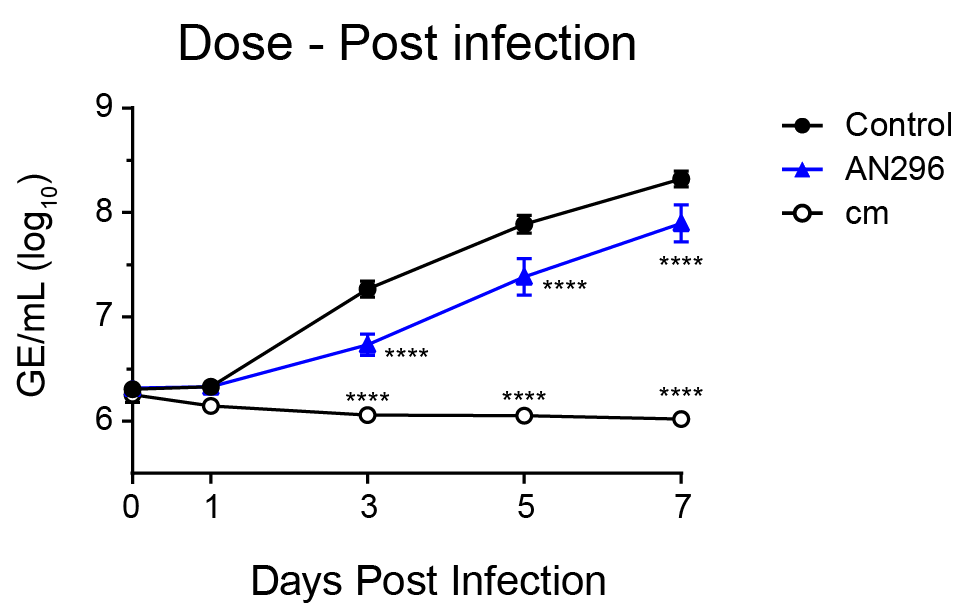

Supplement: S3 Fig — Intracellular replication of C. burnetii NMII in THP-1 cells in the presence of 50 μM AN296 (blue triangle), 31 μM chloramphenicol (Cm) (open circle) or control (closed circle), introduced after the 4 h infection period. Error bars represent standard error of the mean (n ═ 5). ****, p < 0.0001. p values were determined using two-way ANOVA, followed by Dunnett’s multiple comparison post-test. (TIF) [file ppat.1011491.s003.tif]

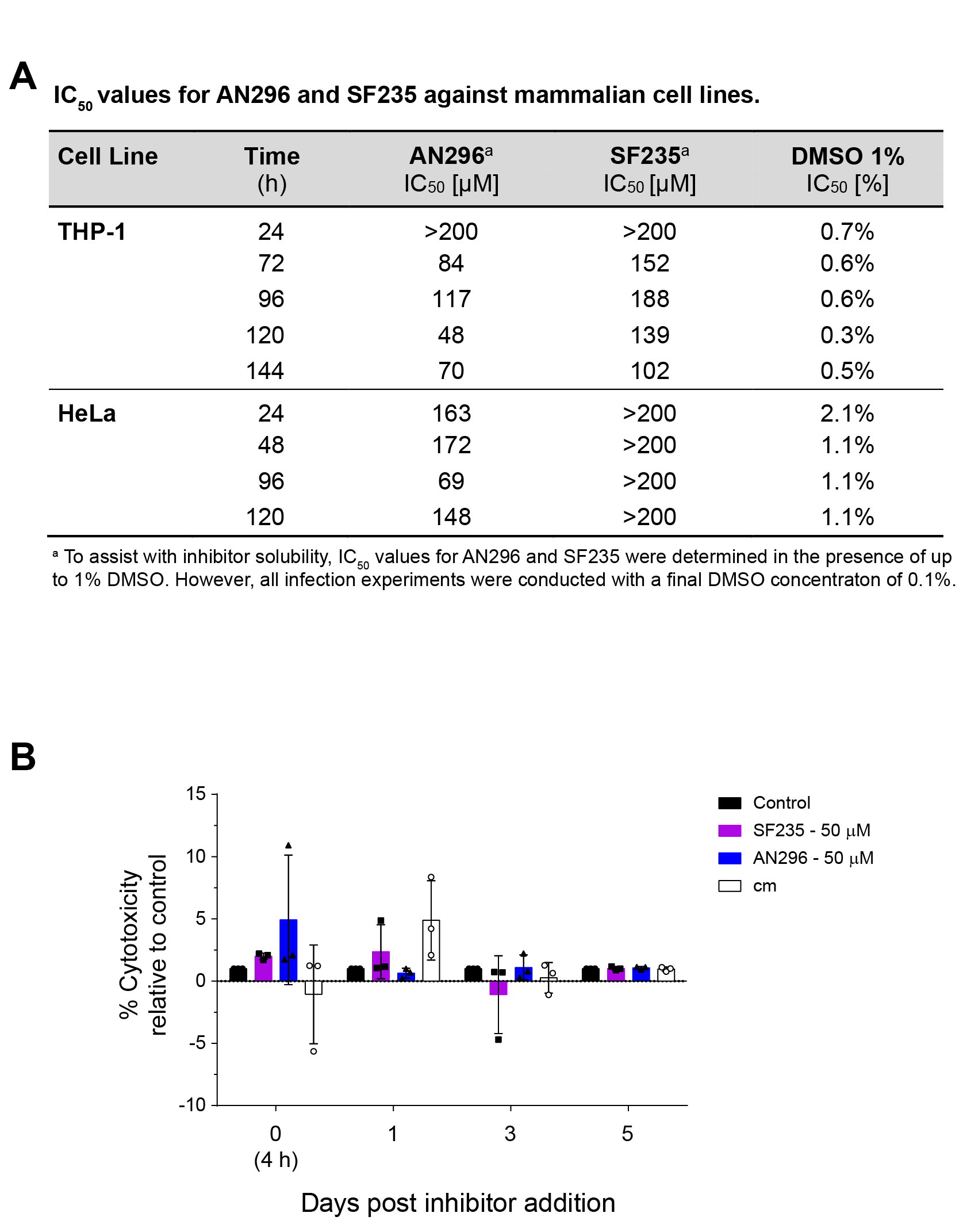

Supplement: S4 Fig — (A) IC50 values for SF235 and AN296 and DMSO after incubation for the indicated periods of time were determined in THP-1 and HeLa cells using the Cell Proliferation Reagent WST-1. (B) Cytotoxicity was measured after THP-1 cells were incubated with SF235 (50 μM), AN296 (50 μM), chloramphenicol (cm, 31 μM) or control (0.1% DMSO) for the indicated period of time using Roche LDH Cell Cytotoxicity kit. No significant difference in cytotoxicity was found between the inhibitors and the control (0.1% DMSO). Data is presented as percent cytotoxicity relative to the control with error bars representing the SD from three independent experiments; p values were determined using two-way ANOVA followed by Dunnett’s multiple comparison post-test. (TIF) [file ppat.1011491.s004.tif]

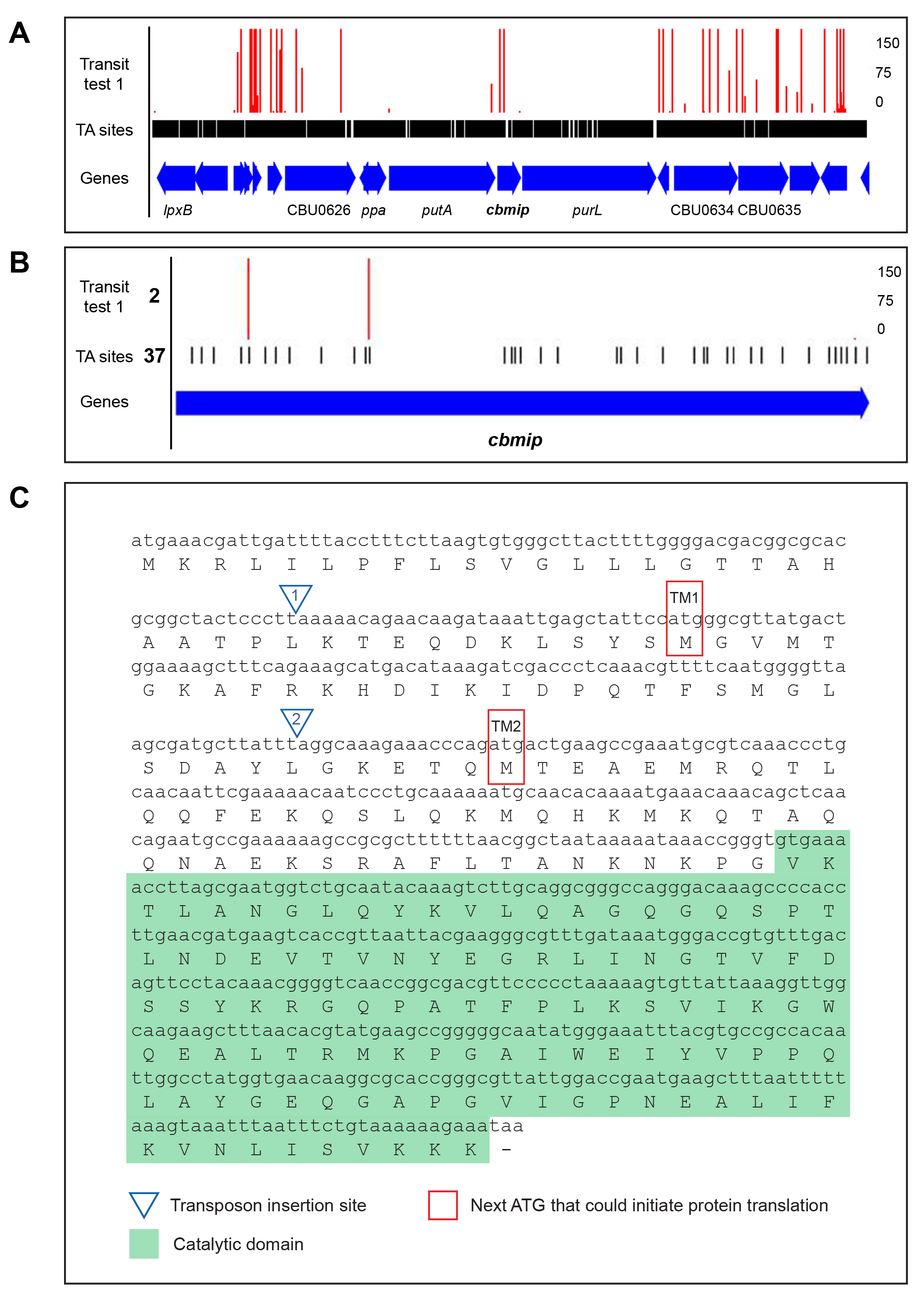

Supplement: S5 Fig — Overview of transposon mutants generated in the Coxiella burnetii NMII Metters et al. library. Confirmed transposon insertion sites are indicated by red vertical lines. All TA insertion sites, possible locations where the transposable element can intergrate, are indicated by black vertical lines. (A) Transposon insertion sites around the cbmip (cbu0630) genetic locus. (B) Enhanced view of transposon insertion sites within the cbmip gene. Two out of a possible 37 mutants were identified in the transposon mutant library. (C) Detailed analysis of transposon insertion sites in cbmip. In lower case text is the DNA coding sequence for the C. burnetii mip gene (cbu0630) of strain C. burnetii NMII. Below, in upper case text, is the encoded amino acid sequence, green box indicates the PPIase catalytic domain. The two transposon insertion sites (TA) identified in the transposon mutant library are indicated by blue triangles. The first ATG following the transposon site is indicated by red boxes. In both instances of transposon insertion in the cbmip sequence, the downstream ATG from which protein translation would initiate is in-frame with the original full-length gene and therefore would result in the production of a truncated CbMip protein in the C. burnetii cbu0630 transposon mutants. (TIF) [file ppat.1011491.s005.tif]

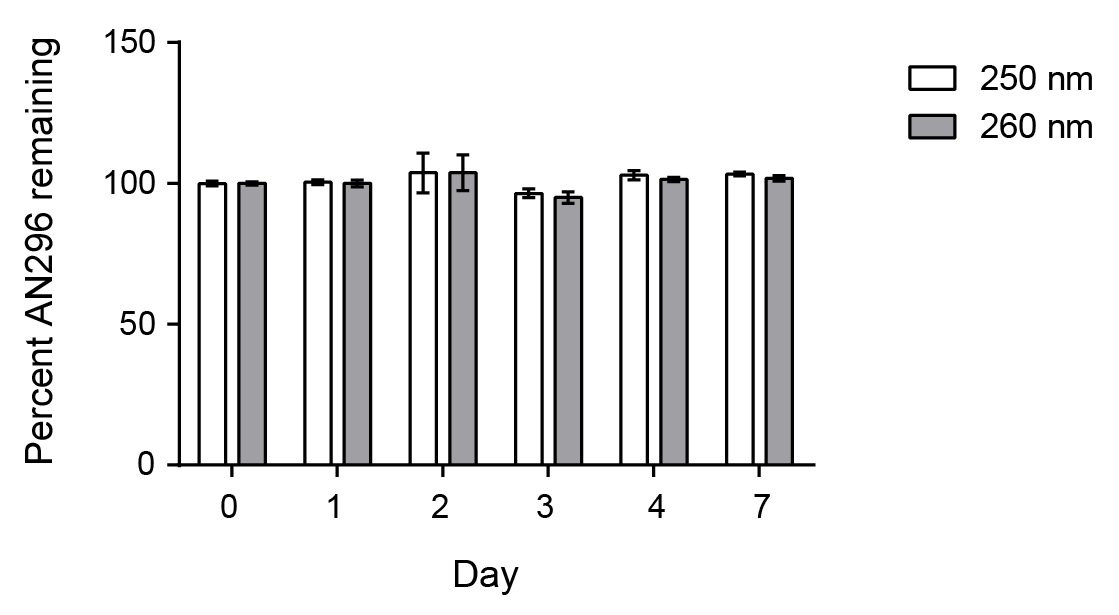

Supplement: S6 Fig — The stability of compound AN296 under acidic conditions (pH of 4.75), was investigated by HPLC chromatography. A stock solution of AN296 (100 μM) was prepared in Buffer C (composition in S4 Table) which mimicked the inorganic components of ACCM-2 media and was incubated at 37°C for a period of 7 days. Data is presented as the amount of AN296 remaining compared to day 0 (at 250 nm and 260 nm), with error bars representing the standard deviation. (TIF) [file ppat.1011491.s006.tif]

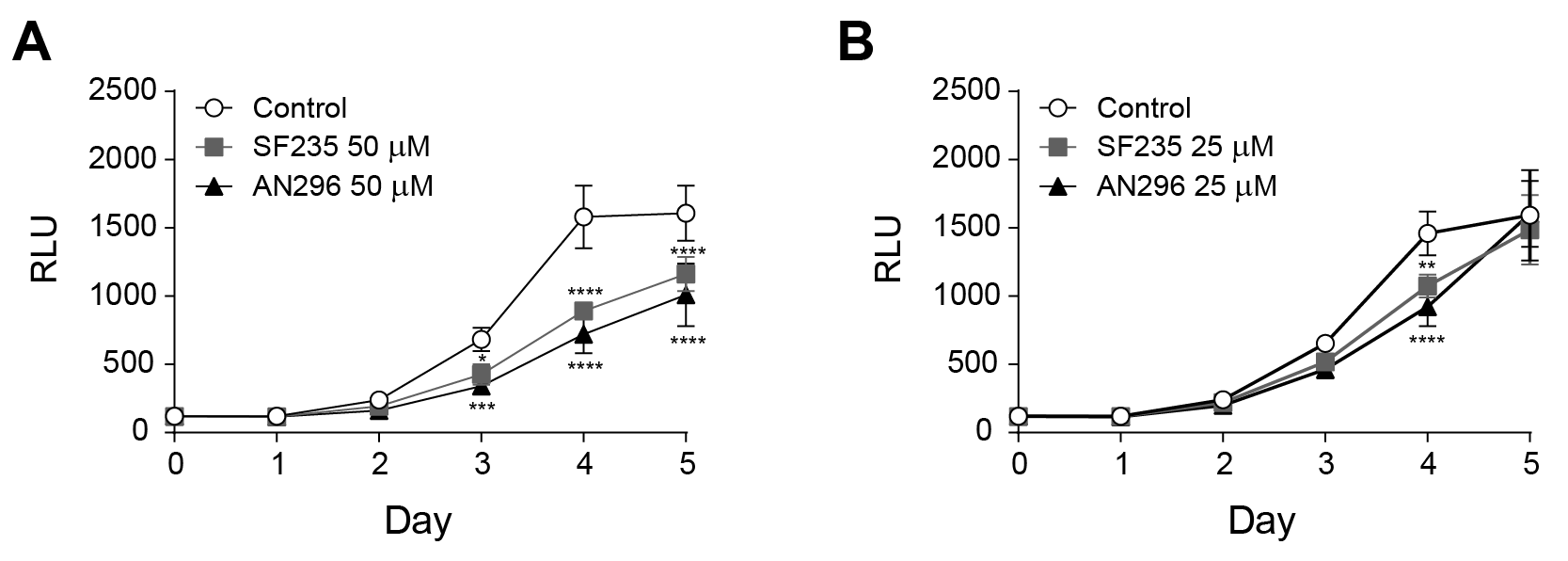

Supplement: S7 Fig — Bioluminescence was measured as an indicator of C. burnetii-lux replication. The strain was inoculated at a concentration of 1 × 10^6^ GE/mL into ACCM-2 media with (A) 50 μM or (B) 25 μM of CbMip inhibitors SF235 (grey square), AN296 (closed triangle) or vehicle control (open circle) and grown over 5 days. Data is presented as RLU (relative light units) with error bars representing the standard deviation from three independent experiments. **, p < 0.01; ***, p < 0.001; ****, p < 0.0001. p values were determined using two-way ANOVA, followed by Dunnett’s multiple comparison post-test. (TIF) [file ppat.1011491.s007.tif]

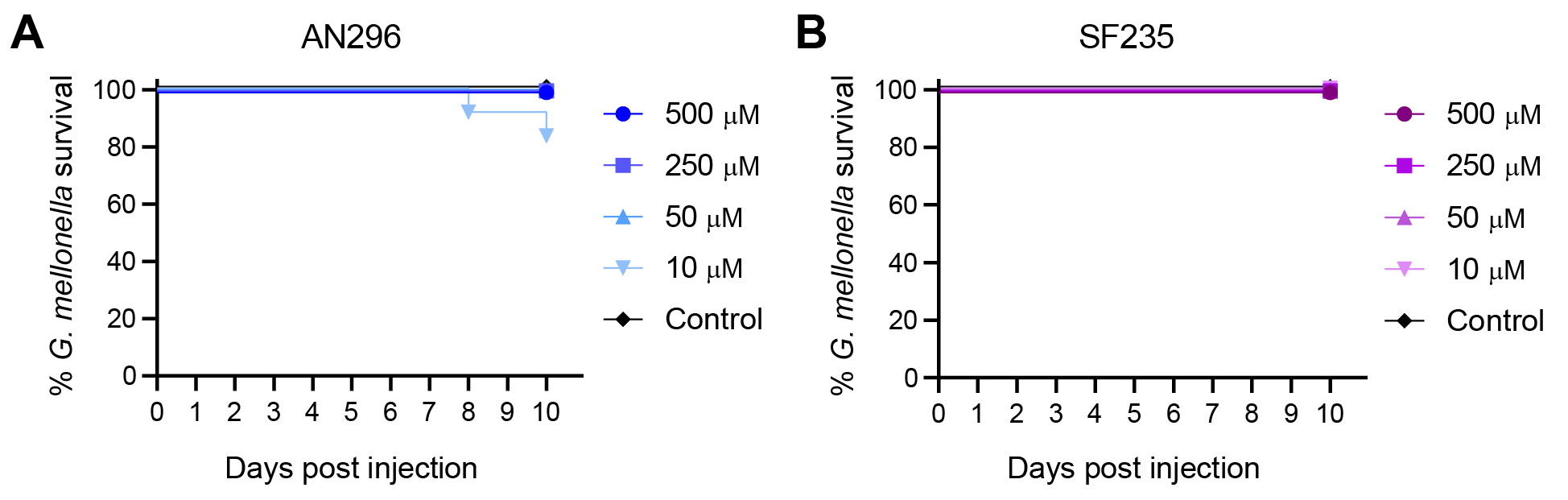

Supplement: S8 Fig — Each larva received a single 10 μL injection of in PBS containing either (A) AN296 or (B) SF235 or vehicle control into the right proleg. Inhibitors were tested at increasing concentrations starting at 10 μM and increasing up to 50 μM, 250 μM and 500 μM (groups of n ═ 10). Larvae were monitored everyday via twitching response over 10 days. All but two larvae, which received 10 μM of AN296, survived the entire duration of the experiment. (TIF) [file ppat.1011491.s008.tif]
