# Supplementary material for: Macrophage infectivity potentiator protein, a peptidyl prolyl cis-trans isomerase, essential for Coxiella burnetii growth and pathogenesis
Source: PLoS Pathog. 2023 Jul 3;19(7):e1011491. doi: 10.1371/journal.ppat.1011491 (PMC10348545; doi:10.1371/journal.ppat.1011491)
Supplement: S1 Table — (DOC) [file ppat.1011491.s009.doc]

**S1 Table. List of strains and plasmids used in this study.**

| **Strain** | **Description** | **Source or reference** |
| --- | --- | --- |
| *E. coli* |  |  |
| TOP10 | F^-^ *mcrA* Δ( *mrr-hsd*RMS-*mcr*BC) Φ80*lac*ZΔM15 Δ *lac*X74 *rec*A1 *ara*D139 Δ( *araleu*)7697 *gal*U *gal*K λ^–^ *rps*L (Str^R^) *end*A1 *nup*G | Invitrogen, Life Technologies (USA) |
| DH5α | F^–^ φ80*lac*ZΔM15 Δ(*lac*ZYA*-argF*)U169 *rec*A1 *end*A1 *hsd*R17 (r_K_^–^, m_K_^+^) *gal*^–^ *pho*A *sup*E44 λ^–^ *thi*-1 *gyr*A96 *rel*A1 | Invitrogen, Life Technologies (USA) |
| BL21(DE3)pLysS | F^–^ *omp*T *hsd*S_B_ (r_B_^–^, m_B_^–^) *gal dcm* (DE3) pLysS(Cam^R^) | Invitrogen, Life Technologies (USA) |
| *C. burnetii* |  |  |
| *C. burnetii* NMI | *C. burnetii* Nine Mile phase I (NMI), strain RSA493, clone 7 | Montana, tick, 1935 |
| *C. burnetii* NMII | Plaque purified *C. burnetii* Nine Mile phase II (NMII), strain RSA439, clone 4 (wild-type). | (1) |
| *C. burnetii*-lux | Luciferase-expressing derivative of wild-type strain *C. burnetii* NMII carrying pTN7-Kan-311-luxCDABE-  TT (Kan^R^) | (2) |
|  |  |  |
| *Plasmids* |  |  |
| pCR-Blunt II-TOPO | High copy cloning vector harbouring Kanamycin/Zeocin resistance. Supplied with Vaccinia virus DNA topoisomerase I. | Invitrogen, Life Technologies (USA) |
| pETM-11 | High copy expression vector facilitating fusion of N-terminal 6xHis-tag followed by a Tev cut site to target protein, Kan^r^ | (3) |
| pMK-cbMipOpt | Synthetic construct from GenScript, containing codon optimised nucleotide sequence of *cbmip*, *mip* gene from *C. burnetii* Nine Mile Phase I, RSA 493, (corresponding to amino acids 22–230) for expression in *E. coli* hosts. |  |
| pETM-11-cbMip | pETM-11 derivative containing nucleotides corresponding to amino acids 22–230 of the *cbmip*, *mip* gene from *C. burnetii* Nine Mile Phase 1 | RSA 493. | This study |
| pETM-11-cbMip-TM1 | pETM-11 derivative containing nucleotides corresponding to amino acids 36–230 of the *cbmip* gene. | This study |
| pETM-11-cbMip-TM2 | pETM-11 derivative containing nucleotides corresponding to amino acids 71–230 of the *cbmip* gene. | This study |
| pJB-Kan | pJB2581 containing *kan* under constitutive expression by the *cbu1169* promoter (*1169^P^*); Kan^r^ | (4) |
| pJC-CAT | pJC84 containing *cat* under constitutive expression by *1169^P^*; Cm^r^ | (5) |
| pJC-CAT::cbu0630-prep | pJC-CAT containing~ 2kb of *cbu0630* 5*′* and 3*′* flanking sequences; Cm^r^ | This study |
| pJC-CAT::cbu0630-Kan | *1169^P^-Kan* cassette cloned into pJC-CAT::*cbu0630-prep;* Cm^r^ Kan^r^ | This study |

**References**

1. Beare PA, Unsworth N, Andoh M, Voth DE, Omsland A, Gilk SD, et al. Comparative genomics reveal extensive transposon-mediated genomic plasticity and diversity among potential effector proteins within the genus Coxiella. Infect Immun. 2009;77(2):642-56.

2. Newton P, Thomas DR, Reed SCO, Lau N, Xu B, Ong SY, et al. Lysosomal degradation products induce Coxiella burnetii virulence. Proc Natl Acad Sci U S A. 2020;117(12):6801-10.

3. Dummler A, Lawrence AM, de Marco A. Simplified screening for the detection of soluble fusion constructs expressed in E. coli using a modular set of vectors. Microb Cell Fact. 2005;4:34.

4. Omsland A, Beare PA, Hill J, Cockrell DC, Howe D, Hansen B, et al. Isolation from animal tissue and genetic transformation of Coxiella burnetii are facilitated by an improved axenic growth medium. Appl Environ Microbiol. 2011;77(11):3720-5.

5. Beare PA, Larson CL, Gilk SD, Heinzen RA. Two systems for targeted gene deletion in Coxiella burnetii. Appl Environ Microbiol. 2012;78(13):4580-9.
