# Supplementary material for: Macrophage infectivity potentiator protein, a peptidyl prolyl cis-trans isomerase, essential for Coxiella burnetii growth and pathogenesis
Source: PLoS Pathog. 2023 Jul 3;19(7):e1011491. doi: 10.1371/journal.ppat.1011491 (PMC10348545; doi:10.1371/journal.ppat.1011491)
Supplement: S2 Table — (DOC) [file ppat.1011491.s010.doc]

**S2 Table. List of oligonucleotides used in this study.**

| **Oligonucleotide** | **Sequence** | **Use** |
| --- | --- | --- |
| OmpA-F | CAGAGCCGGGAGTCAAGCT | Quantification of Coxiella genomes in qPCR |
| OmpA-R | CTGAGTAGGAGATTTGAATCGC |  |
| CbMip_NcoI F | CCCCATGGCCACCCCGCTGAAAACCG | Cloning codon optimized *cbmip* gene, aa 22 -230 |
| CbMip_BamHI R | CCGGATCCTCATTTCTTTTTGACGG |  |
| TM1-CbMIP F | TCCATGGGCGTGATGACCGGCA | Cloning codon optimized *cbmip* gene, aa 36 -230 |
| TM2-CbMIP F | TCCATGGCAATGACGGAAGCGGAAATGCG | Cloning codon optimized *cbmip* gene, aa 71 -230 |
| cbu0630-up F | AAGGATCCTAGCGCAAATGGAAGGACTT | Construction of  pJC-CAT:: cbu0630-prep |
| cbu0630-up R | CATACGGGCTACTGAGGTTGCGGCCGCTCAGCGATCTCCTGAATTATGAGT |  |
| cbu0630-down F | ACTCATAATTCAGGAGATCGCTGAGCGGCCGCAACCTCAGTAGCCCGTATG |  |
| cbu0630-down R | AAGTCGACGGA GAT CGA TAG GGC GAT TAT GA |  |
| P1169-Kan-NotI F | GACGCGGCCGCAGCTTATGGCTTCGTTTCGCAG | Amplification of  *1169^P^*-Kan cassette |
| P1169-Kan-NotI R | GACGCGGCCGCTCAGAAGAACTCGTCAAGAAGGCG |  |
