# Supplementary material for: Macrophage infectivity potentiator protein, a peptidyl prolyl cis-trans isomerase, essential for Coxiella burnetii growth and pathogenesis
Source: PLoS Pathog. 2023 Jul 3;19(7):e1011491. doi: 10.1371/journal.ppat.1011491 (PMC10348545; doi:10.1371/journal.ppat.1011491)
Supplement: S3 Table — (DOC) [file ppat.1011491.s011.doc]

**S3 Table. The codon optimized sequence of *cbmip* and the expected protein sequences of *Cb*Mip and the truncates, *Cb*Mip-TM1 and *Cb*Mip-TM2, for recombinant protein expression.**

|  | Codon optimized nucleotide sequence |
| --- | --- |
| *cbmip* | ATGGGCGCCACCCCGCTGAAAACCGAACAAGATAAACTGTCCTACTCAATGGGCGTGATGACCGGCAAAGCCTTCCGTAAACATGATATTAAAATTGATCCGCAGACCTTTAGTATGGGTCTGTCCGACGCATATCTGGGCAAAGAAACCCAAATGACGGAAGCGGAAATGCGTCAGACCCTGCAGCAATTTGAAAAACAGTCACTGCAGAAAATGCAGCATAAAATGAAACAGACGGCGCAGCAAAACGCCGAAAAATCGCGCGCATTCCTGACCGCTAACAAAAACAAACCGGGTGTCAAAACGCTGGCGAATGGCCTGCAATACAAAGTGCTGCAGGCCGGTCAGGGTCAAAGCCCGACCCTGAACGATGAAGTGACGGTTAATTATGAAGGTCGTCTGATTAACGGCACCGTTTTTGACAGCTCTTACAAACGCGGTCAGCCGGCGACGTTCCCGCTGAAAAGTGTCATCAAAGGTTGGCAGGAAGCACTGACCCGTATGAAACCGGGCGCTATTTGGGAAATCTATGTGCCGCCGCAACTGGCATACGGTGAACAGGGTGCCCCGGGCGTTATTGGTCCGAACGAAGCCCTGATCTTCAAAGTCAACCTGATTTCCGTCAAAAAGAAA |
|  | Protein sequence |
| *Cb*Mip | MKHHHHHHPMSDYDIPTTENLYFQGAMATPLKTEQDKLSYSMGVMTGKAFRKHDIKIDPQTFSMGLSDAYLGKETQMTEAEMRQTLQQFEKQSLQKMQHKMKQTAQQNAEKSRAFLTANKNKPGVKTLANGLQYKVLQAGQGQSPTLNDEVTVNYEGRLINGTVFDSSYKRGQPATFPLKSVIKGWQEALTRMKPGAIWEIYVPPQLAYGEQGAPGVIGPNEALIFKVNLISVKKK |
| *Cb*Mip-TM1 | MKHHHHHHPMSDYDIPTTENLYFQGAMGVMTGKAFRKHDIKIDPQTFSMGLSDAYLGKETQMTEAEMRQTLQQFEKQSLQKMQHKMKQTAQQNAEKSRAFLTANKNKPGVKTLANGLQYKVLQAGQGQSPTLNDEVTVNYEGRLINGTVFDSSYKRGQPATFPLKSVIKGWQEALTRMKPGAIWEIYVPPQLAYGEQGAPGVIGPNEALIFKVNLISVKKK |
| *Cb*Mip-TM2 | MKHHHHHHPMSDYDIPTTENLYFQGAMAMTEAEMRQTLQQFEKQSLQKMQHKMKQTAQQNAEKSRAFLTANKNKPGVKTLANGLQYKVLQAGQGQSPTLNDEVTVNYEGRLINGTVFDSSYKRGQPATFPLKSVIKGWQEALTRMKPGAIWEIYVPPQLAYGEQGAPGVIGPNEALIFKVNLISVKKK |
