# Supplementary material for: Macrophage infectivity potentiator protein, a peptidyl prolyl cis-trans isomerase, essential for Coxiella burnetii growth and pathogenesis
Source: PLoS Pathog. 2023 Jul 3;19(7):e1011491. doi: 10.1371/journal.ppat.1011491 (PMC10348545; doi:10.1371/journal.ppat.1011491)
Supplement: S4 Table — (DOC) [file ppat.1011491.s012.doc]

**S4 Table. Buffer C composition.**

| **Salt** | **mg/L** | **Brand** |
| --- | --- | --- |
| CaCl_2_ | 20 | Sigma-Aldrich Chemie GmbH (Steinheim, Germany) |
| Citric acid*H_2_O | 5,622 | ORG Laborchemie GmbH( Bunde, Germany) |
| Citrat*3Na*H_2_O | 8,863 | Sigma-Aldrich Chemie GmbH (Steinheim, Germany) |
| FeSO_4_*7H_2_O | 491 | Sigma-Aldrich Chemie GmbH (Steinheim, Germany) |
| MgCl_2_*6H_2_O | 406 | Sigma-Aldrich Chemie GmbH (Steinheim, Germany) |
| KH_2_PO_4_ | 1,000 | Merck KGaA (Darmstadt, Germany) |
| NaCl | 14,560 | Carl Roth (Karlsruhe, Germany) |
| NaOH 6N | To pH 4.74 | Sigma-Aldrich Chemie GmbH (Steinheim, Germany) |
| MilliQ |  | Merck Millipore (Schwalbach, Germany) |
